# Immune System Alterations in Multiple Myeloma: Molecular Mechanisms and Therapeutic Strategies to Reverse Immunosuppression

**DOI:** 10.3390/cancers13061353

**Published:** 2021-03-17

**Authors:** Andrea Díaz-Tejedor, Mauro Lorenzo-Mohamed, Noemí Puig, Ramón García-Sanz, María-Victoria Mateos, Mercedes Garayoa, Teresa Paíno

**Affiliations:** 1 Centro de Investigación del Cáncer-IBMCC (CSIC-Universidad de Salamanca), Complejo Asistencial Universitario de Salamanca-IBSAL, Department of Hematology, 37007 Salamanca, Spain; adiaz062@usal.es (A.D.-T.); lorenzomohamed.mauro@usal.es (M.L.-M.); npuig@saludcastillayleon.es (N.P.); rgarcias@usal.es (R.G.-S.); mvmateos@usal.es (M.-V.M.); mgarayoa@usal.es (M.G.); 2 Centro de Investigación Biomédica en Red de Cáncer (CIBERONC, CB16/12/00233), Instituto de Salud Carlos III, 37007 Salamanca, Spain

**Keywords:** multiple myeloma, immune system, immunosuppression, monoclonal antibodies, immune stimulating drugs

## Abstract

**Simple Summary:**

A common characteristic of multiple myeloma (MM) is the dysfunction of patients’ immune system, a condition termed immunosuppression. This state is mainly due to alterations in the number and functionality of the principal immune populations. In this setting, immunotherapy has acquired high relevance in the last years and the investigation of agents that boost the immune system represent a field of interest. In the present review, we will summarize the main cellular and molecular alterations observed in MM patients’ immune system. Furthermore, we will describe the mechanisms of action of the four immunotherapeutic drugs approved so far for the treatment of MM, which are part of the group of monoclonal antibodies (mAbs). Finally, the immune-stimulating effects of several therapeutic agents are described due to their potential role in reversing immunosuppression and, therefore, in favoring the efficacy of immunotherapy drugs, such as mAbs, as part of future pharmacological combinations.

**Abstract:**

Immunosuppression is a common feature of multiple myeloma (MM) patients and has been associated with disease evolution from its precursor stages. MM cells promote immunosuppressive effects due to both the secretion of soluble factors, which inhibit the function of immune effector cells, and the recruitment of immunosuppressive populations. Alterations in the expression of surface molecules are also responsible for immunosuppression. In this scenario, immunotherapy, as is the case of immunotherapeutic monoclonal antibodies (mAbs), aims to boost the immune system against tumor cells. In fact, mAbs exert part of their cytotoxic effects through different cellular and soluble immune components and, therefore, patients’ immunosuppressive status could reduce their efficacy. Here, we will expose the alterations observed in symptomatic MM, as compared to its precursor stages and healthy subjects, in the main immune populations, especially the inhibition of effector cells and the activation of immunosuppressive populations. Additionally, we will revise the mechanisms responsible for all these alterations, including the interplay between MM cells and immune cells and the interactions among immune cells themselves. We will also summarize the main mechanisms of action of the four mAbs approved so far for the treatment of MM. Finally, we will discuss the potential immune-stimulating effects of non-immunotherapeutic drugs, which could enhance the efficacy of immunotherapeutic treatments.

## 1. Introduction

Multiple myeloma (MM), the second most common hematological malignancy, is characterized by the accumulation of malignant plasma cells in the bone marrow (BM) leading to hypercalcemia, bone destruction, anemia and renal failure [1]. Although novel treatments have improved the outcome of MM patients, the disease remains incurable due to continuous relapses increasingly resistant to treatments [2]. Taking into account that MM is strongly influenced by the BM microenvironment [3], treatments should ideally have a dual role, not only on the myeloma cell but also on the microenvironment. In fact, immunomodulatory drugs (IMiDs) have been shown not only to directly attack the tumor, but also to stimulate the immune system [4] through different mechanisms that will be further reviewed.

Although the BM microenvironment is constituted by different components such as, immune cells (i.e., T cells, natural killer (NK) cells, dendritic cells, etc.), non-immune cells (i.e., bone marrow stromal cells (BMSCs), osteoblasts, osteoclasts, etc.), matrix proteins (i.e., fibronectin, laminin, etc.) and secreted soluble factors (i.e., cytokines, growth factors, etc.), the present review will be focused on the major alterations described for immune components in patients with MM. Specifically, we will review how different immune populations together with soluble factors are altered in the context of MM and its precursor stages, leading altogether to an immunosuppressive microenvironment. Finally, we will further address how immunotherapy and immune-stimulating drugs are able to revert this state.

## 2. General Alterations of the Immune System in the Context of Monoclonal Gammopathies

A general alteration of different immune populations and the cytokine profile has been described in patients with monoclonal gammopathies. It is broadly known that in the context of MM, both cell-to-cell contacts, in which myeloma cells, immune cells and other cells from the BM microenvironment are involved, and the presence of different extracellular factors lead to a general immunosuppressive status, which inhibits effector populations and recruits and activates immunosuppressive populations [5].

**Figure 1:**
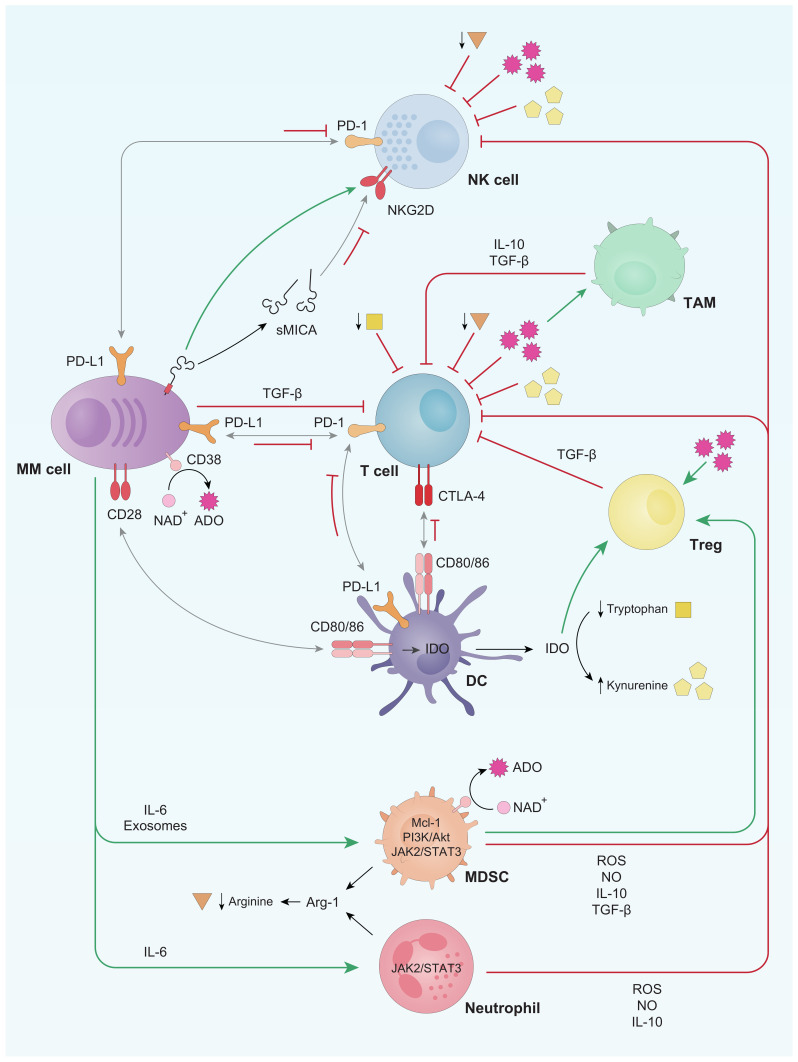
Schematic representation of the main immune system alterations described in multiple myeloma (MM) patients. Briefly, T and natural killer (NK) cells are inhibited through both soluble factors and cell-to-cell contacts with either myeloma cells or other immune cell populations. Grey arrows refer to receptor-ligand union, green arrows stand for activation whereas red bar-headed lines indicate inhibition. Regarding soluble factors, squares refer to tryptophan, pentagons to kynurenine, spiked circles to adenosine (ADO) and triangles to arginine. TAM: tumor associated macrophages; Treg: regulatory T lymphocytes; MDSC: myeloid derived suppressor cells; IDO: indoleamine-2,3-dioxygenase.

Hereunder, we will discuss the alterations described in the number and function of the major immune populations and the molecular factors involved in these variations. These aspects are schematized in Figure 1.

### 2.1. T Cells

T lymphocytes, both T helper CD4^+^ and cytotoxic CD8^+^ cells, are the components of the adaptive immune system that act as coordinators and effectors of immunity [6]. Both subsets play a crucial role in the antitumoral immunity. Regarding MM, the most frequently described alteration in patients is the decrease in CD4^+^ T lymphocyte counts, which is associated with a lower progression free survival (PFS) and overall survival (OS) and a higher relapsing probability [5,7]. Preliminary data from our group show higher percentages of CD4^+^ T cells in the BM of patients with newly diagnosed MM (NDMM) and high risk smoldering MM as compared to healthy subjects, but this was not found in patients with monoclonal gammopathy of undetermined significance (MGUS) [8]. Depending on the immunological context, CD4^+^ T cells can acquire phenotypes with pro or anti-inflammatory functions, named Th1 and Th2, respectively, being the balance of these two cell types important for an efficient immune response [9]. In MM, both increase or decrease in the Th1/Th2 ratio have been observed, therefore making it difficult to elucidate the clinical implications of these findings [10,11,12]. Additionally, the production of TGF- β and IL-6 from MM cells and BMSCs induces the differentiation of naïve T cells to Th17 cells [13], an immunosuppressive CD4^+^ T cell subset, which, as a positive feedback loop, secretes different cytokines promoting MM cell expansion [14].

Regarding cytotoxic T cells, our group has reported an increase in the total number of T CD8^+^ cells in both MGUS and symptomatic MM with respect to healthy donors [15]. More recently Zavidij et al. have found that during disease progression a depletion of memory CD8^+^ T cell subset is observed, thus, being more abundant in healthy and MGUS individuals in comparison to smoldering myeloma (SMM) and active MM [16]. Additionally, an impaired response against viral antigens in MM patients has been observed [17], which may be associated to the increased expression of suppressor of cytokine signaling 1 (SOCS1) by T CD8^+^ subset, which, in turn, inhibits IL-2, IL-6 and IFN-γ production in these same cells, attenuating Th1 and cytotoxic T lymphocytes (CTL)-mediated responses [18]. Soluble factors have also been observed to modulate the activity of cytotoxic T cells. In this sense, TGF-β inhibits IL-2-dependent proliferation and maturation of T cells and prevents naïve T cells from acquiring effector functions [19,20]. The activity of effector T lymphocytes is also inhibited by the immunosuppressive nucleoside adenosine (ADO), derived from ATP or NAD^+^ after sequential catalytic reactions initiated by the surface molecule CD38 in MM cells [21,22,23].

The activation of T cells is initiated through antigen recognition by the T cell receptor (TCR), and then regulated by a balance between costimulatory and coinhibitory signals denominated immune checkpoints [24]. Although immune checkpoints are crucial for the maintenance of self-tolerance and homeostasis [24], the expression of immune checkpoint proteins can be dysregulated by tumors as a mechanism of immune evasion [25]. One of the most relevant immune checkpoints is the programmed-death (PD) pathway. PD-1 is an inhibitory receptor expressed by T cells, which interacts with its ligands PD-L1/PD-L2 expressed by antigen presenting cells (APCs) to inhibit T cell effector functions [26,27,28]. It is known that plasma cells from healthy subjects do not express PD-1 ligands, however, PD-L1/PD-L2 can be found in plasma cells from myeloma patients [29,30,31,32] and in MM cell lines [33]. In addition, our group reported that PD-L1 is also expressed by BMSCs [30]. Moreover, the expression of PD-1 seems to be increased on T cells from MM patients, compared to healthy subjects [34,35], particularly in the setting of relapsed/refractory disease [30,35]. The soluble form of PD-L1, which is released from tumor cells’ surface is also thought to exert immunosuppressive activity [36]. Indeed, high serum soluble PD-L1 levels are associated with poor prognosis in MM patients [37,38] and PD-L1^+^ MM cells show greater drug resistance [39] and higher levels of antiapoptotic proteins [31]. Considering all these data, three main mAbs targeting PD-1 (nivolumab, pembrolizumab and pidilizumab) have been evaluated in MM. Although preclinical murine models showed that PD-1 blockade inhibited tumor growth, both in monotherapy [30,35,40] and in combination [34], data from clinical trials indicate no benefit when used in monotherapy. In fact, our group tested the use of pembrolizumab as consolidation in patients achieving at least a very good partial response (VGPR) but with persistent measurable disease after a first or second line treatment; nevertheless, no upgrades in the quality of the baseline responses could be documented [41]. The combination of pembrolizumab with either pomalidomide or lenalidomide was tested in phase II trials with promising results [42,43]. However, phase III trials of pembrolizumab in combination with the same agents (KEYNOTE-183 (NCT02576977) and KEYNOTE-185 (NCT02579863)) had to be prematurely stopped due to a survival imbalance disfavoring patients receiving the mAb [44,45]. Together with PD-1/PD-L1 pathway, cytotoxic T-Lymphocyte Antigen 4 (CTLA-4) is another immune checkpoint responsible of T cell suppression. The binding of CTLA-4 present in T cells to its ligands (CD80/CD86), expressed on APCs, transmits an inhibitory signal to T cells [46]. In the clinical setting, some studies have assessed the safety and efficacy of CTLA-4 inhibition as consolidation following ASCT in patients with MM. One of them administered ipilimumab and nivolumab between 14 and 28 days post-ASCT in patients with high-risk MM achieving at least stable disease after induction treatment. At 18 months post-ASCT, authors reported a PFS of 71% [47]. The definite niche of checkpoint inhibitors in the treatment of MM patients will be based on the results of clinical trials testing their optimal partners and times of administration.

### 2.2. NK Cells

Natural killer cells (NK cells) are cytotoxic lymphocytes from the innate immune system that take part in the early response to viral antigens and in attacking tumor cells, recognizing and eliminating cells that express stress proteins without needing antigen presentation on major histocompatibility complex (MHC-I) molecules [48]. In fact, activation of NK cells depends on a balance between activating natural cytotoxicity receptors (NCRs) and inhibitory receptors. In humans, activating receptors include NKp30, NKp46, NKp44, DNAM-1 and NKG2D, among others, while inhibitory receptors include killing inhibitory receptors (KIRs) and NKG2A, among others [18]. Briefly, NK cells recognize malignant cells and kill them through secretion of granzyme B and perforin or alternatively, through death signaling pathways in which FasL and TRAIL proteins are involved [49].

In the context of the disease, both MGUS and MM patients present an enrichment in NK cell population in comparison to healthy adults [15,16]. Initially, myeloma cells are sensitive to the lysis induced by NK cells since they express high levels of the stress-induced self-antigen MICA (the ligand of NKG2D receptor). In contrast, as the disease evolves, myeloma cells lose MICA expression and MICA shedding occurs, this latter phenomenon being directly correlated to disease progression [50,51]. In addition, some authors have reported that NKG2D expression was lowered in NK cells from MM patients [51,52,53], while others have not found any differential expression in comparison to healthy donors [54]. Regarding DNAM-1, its expression is reduced on NK cells from myeloma patients with active disease compared to patients in remission or healthy individuals [55]. Furthermore, unlike healthy donors, NK cells from myeloma patients express the PD-1 molecule, which mitigates their functionality even more [35]. It has been reported that NK cell functionality is inhibited by immunosuppressive cytokines found in the tumor milieu, such as ADO, which inhibits NK cell lytic activity [23], or TGF-β, which inhibits the differentiation of functional CD16^+^ NK cells from its CD16^−^ counterparts [56]. Although specific data confirming these findings in the context of MM have not been published, it is possible that similar effects occur since both ADO and TGF-β are increased in the MM microenvironment.

In addition to cytokines, extracellular vesicles (EVs) also have an impact on the behavior of NK cells. In fact, under some circumstances, such as under treatment with sublethal doses of melphalan or doxorubicin, myeloma cells have been found to produce exosomes capable of activating IFN-γ production by NK cells [57] or augmenting NK proliferation and activation [58], thus enhancing NK-cell immune surveillance. However, tumor-derived exosomes have been observed to contain TGF-β, MICA/B, ULBP3, PI-9 and miR-1245 and to contribute to impairment of NK function [59]. In fact, it has been reported that exosomes from myeloma cell lines contain TGF-β and ligands for NK activating receptors, and negatively regulate NK cytolytic ability against MM [60]. In line with this, our group has found that exosomes from BM plasma of myeloma patients similarly reduced the cytolytic activity of normal NKs on myeloma cells, thus contributing to myeloma immunosuppression (unpublished data from our group).

### 2.3. B Cells

B lymphocytes are the components of humoral immunity in the adaptive immune system, which act through antibody secretion [61]. B cell lineage is heavily compromised in MM, with a displaced equilibrium towards a high proliferation of malignant plasma cells. Indeed, MM patients present both a decrease in CD19^+^ B cells [62,63], inversely correlated with disease stage [63], and a reduced ability to secrete polyclonal immunoglobulins and to differentiate into antibody-secreting plasma cells [62]. Moreover, the risk of progression to symptomatic MM from presymptomatic stages (MGUS and SMM) is directly related to the proportion of normal bone marrow plasma cells at diagnosis [64]. Furthermore, the number of B regulatory (Bregs) cells with CD19^+^CD24^high^CD38^high^ phenotype, increases in the transition from MGUS to symptomatic MM [65], supporting the bone marrow milieu by both reducing NK-mediated lysis of MM cells and producing IL-10 [66].

### 2.4. Dendritic Cells (DCs)

Dendritic cells (DCs) are APCs whose main role is the processing of antigenic material, which is then displayed on their cell surface to induce naïve T cell activation. They are classified as plasmacytoid DCs (pDCs), which secrete high levels of type I IFN in response to viral antigens and other stimuli, and myeloid DCs (mDCs) rather involved in antigen presenting and inducing T CD4^+^ and CD8^+^ cell responses [67,68]. The role and general status of DCs in MM is not clear yet. Many studies concluded that DCs from MM patients have impaired T-cell stimulation capacities, whereas contradictory results exist regarding the frequency and phenotype of DCs [69,70,71,72]. Our group demonstrated that the number of BM DCs differed significantly between MM patients with long-term disease control and those with symptomatic disease, with a trend to cell count recovering in the former cohort towards levels similar to those found in healthy adults [15].

DCs are concentrated in the BM during MGUS to MM progression and are able to process and cross-present antigens from apoptotic MM cells via CD91, thus activating myeloma-specific CD8^+^ T cells [73]. Besides, by using their surface CD80/86 molecules, DCs interact with nonapoptotic plasma cells via the overexpressed CD28 receptor, provoking the production of the immunosuppressive enzyme indoleamine-2,3-dioxygenase (IDO) [74], which impairs the immune surveillance through different mechanisms: (i) metabolizing and, therefore depleting, tryptophan from the microenvironment, which is an essential amino acid for T cells, and consequently, producing kynurenine, a toxic compound for T and NK cells [75]; (ii) promoting the development, stabilization and activation of Tregs [76] and (iii) polarizing macrophages and DCs towards an immunotolerogenic phenotype [77]. Along with IDO, ADO is also able to increase the number of tolerogenic DCs [23]. Moreover, pDCs and a percentage of mDCs also express high surface levels of PD-L1 [78], participating in the maintenance of the immunosuppressive bone marrow microenvironment. In addition, TGF-β contributes to the altered immune tumor niche since it inhibits the upregulation of critical T-cell costimulatory molecules on the surface of DCs, reducing their antigen-presenting capacity [79].

### 2.5. Tumor Associated Macrophages (TAMs)

Tumor associated macrophages (TAMs) constitute an abundant component of the myeloma microenvironment that enhances myeloma cell survival and drug resistance through different mechanisms [80]. Within the BM niche, TAMs acquire a secretory profile characterized by a great production of IL-6, IL-10 and proangiogenic factors, such as vascular endothelial growth factor (VEGF), metalloproteinases (MMPs) and cyclooxygenase-2 (COX-2) [81], providing an optimal milieu for myeloma cell growth. Moreover, TAMs resemble a M2-like macrophage population, with little cytotoxicity against tumor cells because of their limited production of nitric oxide (NO) and proinflammatory cytokines, and a poor antigen-presenting capability [82]. Additionally, ADO further polarizes macrophages towards a M2 phenotype [23].

There are several reports describing an association between macrophage infiltration, vascularity and disease prognosis. Suyani et al. showed increased numbers of M2 macrophages in the BM of 68 MM patients, which was associated with unfavorable prognosis and increased microvessel density [83]. Two different studies also reported a negative correlation between CD163 and CD206 expression, which are M2–macrophage markers, and OS in patients with MM [84,85]. Further studies in MM patients confirmed that TAM infiltration in the BM was associated with poor prognosis and drug resistance [86].

There has been shown that the number of M2 macrophages was significantly increased in the BM of MM patients compared with MGUS and SMM, and with healthy donors, suggesting that the malignant plasma cell may be involved in this change to a M2-like phenotype [87]. A very recent single-cell RNA sequencing study revealed that mature CD14^+^ monocytes lose the surface expression of HLA-II molecules as early as in the MGUS stage, resulting in T cell suppression, and suggesting that some of these sequential immune alterations begin on an early stage of the disease [16].

### 2.6. Myeloid-Derived Suppressor Cells (MDSCs) and Neutrophils

Myeloid-derived suppressor cells (MDSCs) are a heterogeneous group of immature myeloid cells endowed with the capacity to suppress the activation, proliferation and cytotoxic capacity of effector T and NK cells. In humans there are two subsets of MDSCs; granulocytic-MDSCs (G-MDSCs) (also called polymorphonuclear, PMN-MDSCs) and monocytic-MDSCs (M-MDSCs) [88]. In myeloma the G-MDSC constitutes the predominant MDSC population in BM and peripheral blood (PB) as opposed to M-MDSC [89]. MDSCs mainly suppress T cell responses by producing reactive oxygen species (ROS) and high amounts of NO, arginase-1 (Arg-1), and immunosuppressive cytokines such as IL-10. There is an increase of G-MDSCs in both PB [90,91] and BM [89,92] of patients with active MM, compared with samples from MGUS or healthy donors. Moreover, it has also been shown that MDSCs from MM patients were able to induce higher Treg differentiation than those from healthy age-matched donors [89]. In vitro data indicate that MDSCs support MM progression by inhibiting effector cells, enhancing Treg development [93], and even by differentiating into osteoclasts [94]. In addition, MM cells promote the survival of MDSCs through Mcl-1 upregulation [95], secretion of IL-6, which drives MDSCs expansion by the activation of several molecular cascades such as PI3K/Akt or JAK/STAT3 [96,97], and through the accumulation of high levels of ADO by the conversion of NAD^+^ [23,98]. Together with cytokines, Wang et al. showed that both BMSC-derived and myeloma-derived exosomes promoted the proliferation and survival of MDSCs [99]; upon incorporation of exosomes, MDSCs also increased their NO production, thus contributing to T cell inhibition [100].

Neutrophils are the most abundant white blood cell in PB, and are essential for clearance of extracellular pathogens, both by direct toxicity and by establishing interactions with other immune cells [101]. In the context of MM, neutrophils present functional defects, such as a reduction in lysozyme activity and an increased secretion of Arg-1 therefore presenting an immunosuppressive behavior [102,103]. In fact, MM patients have increased serum levels of Arg-1 [102], which depletes arginine on tumor microenvironment, an essential amino acid for T and NK cell proliferation [104]. Furthermore, as disease evolves from MGUS to MM, neutrophils progressively activate the JAK-2/STAT3 pathway in response to MM cell exposure, which further supports the immunotolerogenic niche due to the production of proinflammatory and survival signals [105]. Interestingly, the neutrophil to lymphocyte ratio (NLR) at diagnosis is able to predict both the outcome in NDMM patients treated with novel agents [106], and the prognosis in patients at day +100 post stem cell autologous transplant [107].

### 2.7. Regulatory T Lymphocytes (Tregs)

Regulatory T lymphocytes (Tregs) are a subpopulation of T cells that modulate the immune system, maintain tolerance to autoantigens and prevent autoimmune reactions. They present a CD4^+^CD25^+^ phenotype and an increase in FOXP3 factor, which is determinant for the development of this subpopulation [108]. They exert their immunosuppressive activity through cell-to-cell contact, secreting immunosuppressive cytokines such as TGF-β and IL-10 [109] or inducing the expression of IDO in DCs, which induces a positive loop since IDO promotes the expansion of Tregs [110]. In addition to IDO, ADO also promotes the expansion of Tregs [23]. Furthermore, in BM samples from NDMM patients, CTLA-4 appears to be overexpressed (along with FOXP3) in Tregs, which suggests a local accumulation of Tregs in the tumor microenvironment [111]. In fact, it has been described that CTLA-4 induces Treg expansion and induction of immunosuppressive cytokines in this population [46].

Most authors have reported that MM patients have a higher Treg percentage [16,112,113,114,115] in comparison to healthy donors, suggesting that myeloma cells escape from the immune system at least partially through the increase of this population. Indeed, recent work suggests that this immune scape occurs early in disease development, since it has already been described in patients with SMM [16]. Interestingly, our group reported that the number of Tregs was lower in patients with long-term disease control than in those with symptomatic MM [15]. In addition, Treg number can be used as a biomarker of disease progression, since patients with higher Treg percentage presented a lower OS [112,116].

## 3. Currently Approved Immunotherapeutic Treatments in MM

**Figure 2:**
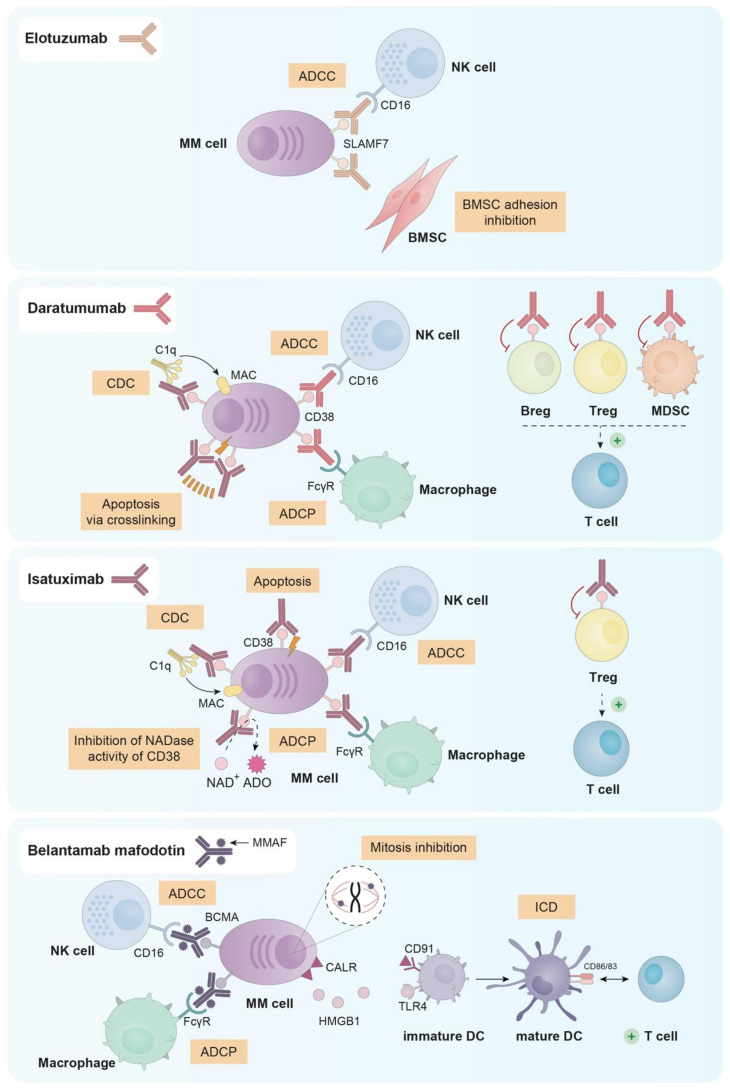
Principal mechanisms of action of the three naked mAbs (elotuzumab, daratumumab and isatuximab) and the ADC (belantamab mafodotin) approved for the treatment of MM.

Monoclonal antibodies (mAbs) have emerged as a backbone therapy for many B-cell tumors, due to their high efficacy and good tolerability. However, the development of effective mAbs for the treatment of MM has been tough, since the discovery of target molecules unique for all MM cells resulted challenging. Up to date, there are three naked mAbs and one antibody–drug conjugate (ADC) approved for the treatment of MM. Their main mechanisms of action can be found in Figure 2.

### 3.1. Elotuzumab

Elotuzumab (anti-SLAMF7) was the first mAb approved by the US Food and Drug Administration (FDA) for the treatment of MM. In particular, elotuzumab was first approved in combination with lenalidomide and dexamethasone for relapsed/refractory myeloma patients who had received one to three prior therapies [117]. SLAMF7, also known as CS1, is a cell surface molecule expressed in plasma cells, CD8^+^ cytotoxic T lymphocytes, activated B cells, NK cells and mature DCs [118,119,120,121]. In the context of MM, SLAMF7 is expressed in both primary malignant plasma cells and in almost all MM cell lines. In addition, soluble SLAMF7 has been detected in serum of MM patients presenting a direct correlation with disease stage [118,121].

Elotuzumab is a humanized IgG1 mAb that inhibits MM cell adhesion to BMSCs, which may reverse the protective effect provided by the bone marrow microenvironment to myeloma cells. Additionally, elotuzumab is able to induce antibody dependent cellular cytotoxicity (ADCC) mediated by NK cells in both MM cell lines and primary plasma cells from myeloma patients (either newly diagnosed or resistant to conventional therapies) [121].

Since NK cells and a small subset of activated lymphocytes express SLAMF7, elotuzumab is able to activate ex vivo different subsets of peripheral blood mononuclear cells (PBMCs) from myeloma patients and healthy donors. Indeed, elotuzumab selectively activated the subpopulation of CD56^dim^ NK cells, upregulating CD69, CD11b and CD54 and downregulating CD16 expression and resulting in the killing of myeloma cells via a CD16-independent mechanism [122]. Moreover, elotuzumab also activated monocytes as evidenced by the up-regulation of SLAMF7, HLA-DR and CD54 [123].

### 3.2. Daratumumab

Daratumumab is an anti-CD38 mAb that was approved in 2015 by the FDA for MM patients who had received at least three prior lines of therapy or for patients double refractory to proteasome inhibitors and immunomodulatory agents [124,125]. Besides, it has been recently approved for NDMM patients ineligible for stem-cell transplantation [126]. CD38 is expressed in different cell subsets from hematopoietic and non-hematopoietic lineages. Regarding the first, CD38 is expressed in Tregs, circulating monocytes, CD4^+^ and CD8^+^ T cells, NK cells, granulocytes/neutrophils, B cell precursors and in terminally differentiated plasma cells from healthy donors [127,128,129]. In the context of MM, CD138^+^ malignant plasma cells express higher levels of CD38 than other immune subsets and normal plasma cells [130]. Moreover, CD38 is also expressed by osteoclasts in the tumor niche [131].

Daratumumab was first selected from a panel of 42 human anti-CD38 mAbs for being effective in killing MM cells via complement dependent cytotoxicity (CDC) and ADCC [132]. Further studies in vitro, ex vivo and in vivo demonstrated that daratumumab was also able to induce programmed cell death in the presence of crosslinking agents (both F(ab)_2_ fragments and Fcγ receptor-expressing cells) [133], and antibody dependent cellular phagocytosis (ADCP) [134].

Given that different immune cell subsets express CD38, daratumumab treatment has an impact on them. In fact, it has been described that MM patients treated with daratumumab both in monotherapy and in combination with lenalidomide and dexamethasone, present a decrease in absolute cell count in NK cells (from 10% to 2%), MDSCs, Bregs and Tregs. On the contrary, other immune populations, such as CD4^+^ and CD8^+^ T cells showed increased numbers [135,136,137,138]. Despite the decrease in NK cell number observed after daratumumab treatment, according to Casneuf et al. the remaining NK cells seemed to be able to contribute to the clinical efficacy of the drug [137]. Furthermore, daratumumab has been reported to induce NK cell activation and degranulation as observed by the upregulation of CD69, CD107a and IFN-γ in this cell subset [139].

### 3.3. Isatuximab

Isatuximab is a humanized IgG1 anti-CD38 mAb that has been recently approved (March 2020) in combination with pomalidomide and dexamethasone for MM patients who had previously received at least two lines of therapy [140]. Isatuximab exerts its antimyeloma effect through different mechanisms. First, and unlike daratumumab, isatuximab has shown proapoptotic activity against myeloma cells expressing high levels of CD38 without any cross-linking agents [141,142]. Moreover, isatuximab also presents immune-mediated cytotoxic effects, such as, the induction of strong CDC, potent ADCC and ADCP [141,143]. In contrast to daratumumab, isatuximab completely inhibits the NADase activity of CD38, which may mitigate the immunosuppressive microenvironment in the bone marrow of MM patients [144,145,146].

As observed with daratumumab, isatuximab is able to suppress Tregs. In fact, in PBMCs from both healthy donors and MM patients treated with isatuximab in vitro, the percentage of Tregs was reduced while the percentage of effector T cells increased. This reduction of Treg frequency was more significant in cells from MM patients than from healthy donors, probably due to the higher expression of CD38 observed in patients’ Tregs [127]. Furthermore, isatuximab upregulated the activation molecules CD107a and IFNγ in monocytes, CD8^+^ T cells and NK cells not only from healthy donors but also from myeloma patients, augmenting the cytotoxic functions of these three cell subsets both in the presence and in the absence of CD38^+^ target cells [127,129]. Additionally, Moreno et al. observed that isatuximab depleted in vitro CD38^high^ B-lymphocyte precursors, basophils and NK cells [143]. In fact, the NK cell depletion observed after isatuximab treatment seems to be generated through activation followed by exhaustion of these cells [143].

### 3.4. Belantamab Mafodotin

Belantamab mafodotin (GSK2857916) is an afucosylated, humanized IgG1 anti-B-cell maturation antigen (BCMA) mAb conjugated with monomethyl auristatin F (MMAF), which is a tubulin polymerization inhibitor [147]. Both parts (anti-BCMA antibody and MMAF toxin) are linked through a non-cleavable maleimidocaproyl linker, which provides better plasma stability of the compound without losing any property and without any nonspecific toxicity [148]. Belantamab mafodotin is the first anti-BCMA ADC approved by the FDA as a single agent for relapsed/refractory multiple myeloma (RRMM) patients who have received at least four prior therapies [149]. BCMA, also known as TNFRSF-17, is selectively induced during plasma cell differentiation being almost absent on naïve and memory B cells [150,151]. BCMA is expressed by several myeloma cell lines [152] and BCMA mRNA is commonly expressed at high levels in primary malignant plasma cells [153].

Belantamab mafodotin exerts its antimyeloma effect through four known mechanisms: (i) ADCC mediated by NK cells; (ii) recruitment of macrophages to promote ADCP; (iii) disruption of microtubules and subsequent G_2_/M cell-cycle arrest followed by apoptosis after the release of the MMAF toxin in the cytoplasm of myeloma cells [147] and (iv) induction of immunogenic cell death (ICD) [148], which is a mechanism characterized by the ability of dying cells to elicit robust adaptive immune responses against altered self-antigens or cancer-derived neo-epitopes [154]. In relation to the latter mechanism, preliminary data indicates that treatment of myeloma cells with belantamab mafodotin promotes the exposure of calreticulin (CALR) on their surface and the release of HMGB1, which subsequently induce the maturation and activation of DCs and eventually the activation of T cells [148].

## 4. Drugs with Immune-Stimulating Activity in Multiple Myeloma

As explained before, some of the mechanisms of action of mAbs require the presence of different immune effector cell subsets. Therefore, agents with immune-stimulating effects could be good partners of mAbs. Next, we will explain the effects on the immune system of different drugs that are currently combined with mAbs in the clinic and some others, which could be postulated as appropriate candidates in new combinations with mAbs. A summary of these effects can be found in Table 1.

### 4.1. Immunomodulatory Drugs (IMiDs)

IMiDs are a class of immunomodulatory drugs with pleiotropic effects on myeloma cells and other immune cells, for which antiangiogenic, cytotoxic and immunomodulatory activities have been reported [4]. Currently, there are three types of IMiDs approved for the treatment of MM namely, thalidomide and its analogues, lenalidomide and pomalidomide and their immunomodulatory properties have been widely described.

Different authors have reported that T cells increase their cytokine production after IMiD exposure. Indeed, Haslett et al. observed the enhancement of cytotoxic and proliferative responses by T cells, mainly by the CD8^+^ subset, and the increase in IL-2 and IFN-γ production promoted by thalidomide [155]. Conversely, Schafer et al. showed that lenalidomide and to a greater extent pomalidomide, increased IL-2 production by both CD4^+^ and CD8^+^ T cells, with a slightly more potent effect on the CD4^+^ subpopulation [157]. In the same work, pomalidomide and lenalidomide enhanced AP-1 transcriptional activity in stimulated T cells, finding that the proximal AP-1 binding site of the IL-2 promoter is involved in the IMiD response [157]. Besides this, pomalidomide promoted the nuclear translocation of NFAT2 via PI3K and its subsequent binding to the IL-2 promoter, further enhancing IL-2 transcription [158]. Franssen et al. provided additional mechanisms for their activity on immune populations, specifically the decrease in the cereblon substrate proteins Ikaros and Aiolos in CD4^+^ T cells, CD8^+^ T cells, NK cells and B-cells. This explained the increase in activated T cells in lenalidomide-refractory patients after treatment with lenalidomide combined with low-dose cyclophosphamide and prednisone [159]. Moreover, these authors also found that pretreatment of PBMCs with lenalidomide enhanced PBMC-mediated killing of both lenalidomide-sensitive and lenalidomide-resistant myeloma cell lines [159].

IMiDs have also been shown to increase NK cell cytotoxic activity [156,158,160,167]. Firstly, this effect was suggested to occur indirectly via induction of IL-2 production in T cells [156,158,160]. However, Hideshima et al. have recently described the phosphorylation and activation of Zap-70 and the involvement of cereblon as two direct and independent mechanisms of pomalidomide-mediated upregulation of granzyme-B expression and NK cell activity [167]. Additionally, other authors observed that IMiDs may enhance the susceptibility of myeloma cells to NK cell-mediated recognition and killing by increasing the expression of MICA and PVR (the ligand for DNAM-1 receptor) in myeloma cells [161]. Specifically, these authors observed that IMiDs increase mRNA expression and promoter activity of MICA and PVR, and suggested that IMiDs may shift both Ikaros and Aiolos depletion and IRF4 downregulation into increased MICA expression, indicating that all these transcription factors repress MICA gene expression [161].

Activation of T and NK cells by IMiDs has also been explained by indirect mechanisms. In this sense, lenalidomide has been reported to reduce PD-L1 expression on RPMI-8226 and primary MM cells, and the combination of lenalidomide and pidilizumab (anti-PD-1) significantly enhanced NK-cell cytotoxicity against myeloma cells [35]. Fujiwara et al. found that pomalidomide treatment promoted tumor killing by CTLs through inhibition of IFN-γ-induced PD-L1 expression in different tumor cells, including myeloma cells [162]. Contrary to these findings, other authors did not observe any reduction of PD-L1 expression after lenalidomide treatment in different MM cell lines [163].

IMiDs have also been proposed to increase anti-tumor immunity by enhancing DC function [164]. Both pomalidomide and lenalidomide increase the endocytic activity of DCs and the expression of MHC Class I and CD86 in these cells [164]. Moreover, when DCs were treated with pomalidomide and lenalidomide prior to antigen presentation assay, both IMiDs effectively increased CD8^+^ T-cell cross-priming, but only pomalidomide was effective in increasing CD4^+^ T-cell priming [164].

Furthermore, lenalidomide and pomalidomide strongly inhibited Treg proliferation and had an inhibitory effect on the suppressor function of these cells, which was associated with decreased FOXP3 expression [165]. Other authors observed that lenalidomide reduced Treg differentiation by myeloma cells due to ICOSL downregulation, whereas dexamethasone induced apoptosis of Tregs, therefore identifying for the first time an immune synergism that explained the observed immune-modulation associated with the lenalidomide + dexamethasone combination therapy [166].

Finally, the IFN-regulated gene *CD38* is repressed by Ikaros and Aiolos, and treatment with lenalidomide increased surface expression of CD38 in several MM cell lines leading to higher efficacy of ADCC mediated by daratumumab [168].

### 4.2. Proteasome Inhibitors (PIs)

The use of PIs, such as bortezomib, carfilzomib and ixazomib, has been incorporated into several regimens for the treatment of MM [189]. In addition to directly induced tumor cell death [190], PIs can exert ICD. In this sense, Chang et al. examined the generation of immune-mediated antitumor effects in response to bortezomib in a murine ovarian tumor model [169]. Treatment with bortezomib resulted in a higher recruitment of CD8^+^ T lymphocytes into the tumor and higher amounts of tumor-infiltrating IFN-γ^+^ T lymphocytes. Moreover, in vitro treatment of ovarian tumor cells with bortezomib led to the surface upregulation of Hsp60 and Hsp90, two ICD markers, which promoted the phagocytosis of tumor cells by DCs [169]. Regarding MM, the delivery of an activating signal from bortezomib-killed myeloma cells to DCs is mediated by the exposure of Hsp90 on the surface of apoptotic cells [170]. Indeed, DCs pulsed with bortezomib-killed myeloma cells are potent inducers of tumor-specific IFN γ–producing T cells [170]. Both bortezomib and carfilzomib promoted in myeloma cell lines the exposure of CALR, another protein marker of ICD [171]. Finally, combined treatment of carfilzomib and chloroquine (which blocks autophagy) increased both apoptosis and cell surface exposure of CALR, therefore increasing the immunogenic ability of carfilzomib [171].

### 4.3. Histone Deacetylase Inhibitors (HDACi)

HDACi exert antimyeloma effects through multiple mechanisms of action including epigenetic, protein stabilizing and immunogenic effects [191], although data regarding the latter are still contradictory and controversial as exposed below.

Moreno-Bost et al. observed that the sequential treatment of MM cells with 5-azacitidine followed by the HDACi MGCD0103 (mocetinostat) increased their susceptibility to the specific lysis mediated by MAGE-A3-specific CTLs and the secretion of IFN-γ by the latter [172]. In other study, valproic acid (VPA) induced the upregulation of MICA/B and ULBP2 in MM cell lines and patients’ myeloma cells, and, consequently, degranulation and cytotoxic activity of NK cells were enhanced in presence of VPA-pretreated myeloma cells [173]. Additionally, sodium butyrate, another HDACi, also upregulates MICA in MM cell lines when combined with a matrix metalloproteinase inhibitor III and phenylarsine oxide, a drug that hinders surface ligand internalization [174]. Moreover, the cytotoxic efficacy of cytokine-induced killer (CIK) cells in targeting myeloma is higher when MM cells are pretreated with a combination of these three drugs [174]. Panobinostat, a pan-HDACi approved for the treatment of relapsed MM, also upregulates ULPBP2/5/6 and MICA/B in MM cells [163].

Regarding effects on the PD-1/PD-L1 axis, the HDAC6 selective inhibitor ACY-241 significantly decreases PD-L1 expression on pDCs, which in turn attenuates PD-L1/PD-1-mediated NK suppression and enhances NK cell-mediated MM cell cytotoxicity [177]. Furthermore, combined treatment of ACY-241 and anti-PD-L1 triggered a more robust cytolytic activity and degranulation against MM cells than each agent alone [177]. With respect to PD-L1 expression in MM cells, panobinostat, entinostat (class I HDAC-specific inhibitor) and ricolinostat (HDAC6 inhibitor) upregulated PD-L1 in these cells probably by histone acetylation of the *PDL1* gene promoter [163]. In line with these results, reanalyzing gene expression microarray data generated in our lab [192], we have found an increase in PD-L1 mRNA expression after treatment of MM.1S cells with panobinostat. On the contrary, Bae et al. found that ACY-241 decreased PD-L1 expression on CD138^+^ tumor cells [178]. ACY-241 also reduced PD-L1 expression on Tregs and PD-1 expression on CD3^+^ T cells, upregulated CD80/86 and MHC molecules (class I and II) on both tumor and DCs and upregulated costimulatory and activation molecules on antigen-specific CTLs [178]. Panobinostat, however, has been shown to impair DC function to stimulate antigen-specific immune responses [176].

Interestingly, several HDACi upregulate the expression of CD38 [175,179,180]. In ex vivo cultures, panobinostat and, to a greater extent, ricolinostat upregulated CD38 expression in myeloma cells from both newly diagnosed and relapsed/refractory patients, which improved the cytotoxic effects of daratumumab [175,179]. Specifically, the inhibition of HDAC6 by ricolinostat prevents the deacetylation of H3K27 in the *CD38* promoter [179]. Moreover, second-generation HDAC6 inhibitors such as ACY-241 and WT-161 also increase CD38 expression in MM cells [179]. Importantly, the class I HDAC-specific inhibitor entinostat enhances CD38 expression alone and in combination with IFN-α, ATRA or both. However HDAC6 inhibition impairs the upregulation of CD38 in MM cells by IFN-α and ATRA, which constitutes an aspect to take into account when considering the possibility of adding a HDACi to the combined treatment of daratumumab, IFN-α and ATRA [180].

### 4.4. Cyclophosphamide

The alkylating agent cyclophosphamide has been used in the treatment of MM for over 60 years and, at low doses, it also presents significant immunomodulatory activity [193]. In this sense, treatment of different tumor models with mafosfamide (a chemical compound related to cyclophosphamide) or cyclophosphamide induces CALR translocation [181,182] and the release of HMGB1 [181], both of them being surrogate ICD markers. Accordingly, cyclophosphamide-treated mice showed an increase in tumor infiltrating DCs with an activated phenotype [181]. Moreover, type I IFN, a cytokine known to stimulate DC activation and T-cell priming, has a synergistic antitumor effect with cyclophosphamide [181]. In addition to ICD induction, cyclophosphamide also depleted Tregs [182,183], promoted Th1 polarization [183], increased activated NK cells [182] and modulated the myeloid population [182] in different tumor models.

In MM, there is still no clear evidence of the involvement of ICD mechanism in the in vivo responses to cyclophosphamide, although other immune-modulating effects have been observed. Thus, in vitro exposure of MM.1S cells to low doses of cyclophosphamide leads to a secretory response, which, along with downregulation of the “don’t eat me” antigen CD47, greatly augments macrophage induced ADCP of daratumumab-coated MM cells [184]. Additionally, after cyclophosphamide treatment, macrophages presented increased levels of the CD64 Fcγ receptor, required for ADCP, possibly further enhancing phagocytosis [184]. These results have been confirmed in the clinical setting in the CyBorD-DARA trial [194].

### 4.5. Arginase Inhibitors

As mentioned previously, Arg-1 exerts immunosuppressive effects and, accordingly, several arginase inhibitors have demonstrated beneficial effects, especially in solid tumors [195]. In MM, however, there are still few studies that have explored the effect of arginase inhibitors. It has been described that the efficacy of bortezomib against MM cells was reduced in presence of either serum obtained from MM patients or conditioned media from MDSCs from MM patients, being both effects reverted by treatment with arginase inhibitors [102]. In other study, two Arg-1 inhibitors, nor-NOHA and CB-1158, reverted the immune-suppressive properties of both MGUS and MM-high-density neutrophils (HDNs) [103]. Contrary to the detrimental effects of arginase, it has also been published that arginase produced by activated macrophages may inhibit the growth of tumor cells [196]. In line with this, Th2 adoptive cell therapy eradicated myeloma cells in a murine model, which was associated with massive infiltration of M2-type macrophages producing arginase and was strongly inhibited by treatment with the arginase inhibitor BEC [185].

### 4.6. IDO Inhibitors

The effects of IDO inhibitors against MM are still controversial. The induction of high IDO production by DCs suppresses effector T cell activation, whereas treatment with the highly selective IDO inhibitor INCB014943 significantly reverses these effects [74]. Furthermore, it has also been observed that IDO is functionally expressed in MM cells [186]. In this sense, IDO^+^ myeloma cells induce an expansion of the overall Treg population and reduce the percentage of IL-2 and IFN-γ-expressing T cells, being both effects partially reverted by D,L-1-methyl-tryptophan (1-methyl-DL-Trp), a chemical inhibitor of IDO [186]. Conversely, Pfeifer et al. found that IDO is expressed in myeloma cells in a low degree and is not upregulated after treatment with the cytokines IFN-γ, HGF and TNF-α [197]. However, IFN-γ stimulation of mesenchymal stromal cells (MSCs) specifically induced IDO in these cells, which may directly induce the apoptosis of myeloma cells by tryptophan depletion or the accumulation of tryptophan metabolites, being these effects abrogated by specific IDO inhibitors like 1-methyl-DL-Trp [197].

Interestingly, IDO is significantly upregulated during osteoclastogenesis [187]. Moreover, expression of IDO in osteoclasts is further enhanced following INF-γ stimulation [187]. Accordingly, both T-cell proliferation and cytotoxic capacity of CTLs are significantly inhibited in presence of osteoclasts and these effects are partly overcome by treatment with the IDO inhibitor 1-methyl-DL-Trp [187].

IL-32γ derived from MM cells significantly induced the production of IDO in macrophages, a phenomenon predominantly dependent on the STAT3 and NF-κB pathways [188]. IDO produced by IL-32γ-educated macrophages inhibits proliferation and effector function of CD4^+^ T cells, being T cell proliferation almost completely restored by adding 1-methyl-DL-Trp [188].

## 5. Concluding Remarks

Due to the wide range of all the immune alterations described so far in monoclonal gammopathies, and also considering the contradictory results in some cases, it is still difficult to accurately predict which patients with MGUS or SMM will progress to active MM based on such alterations. Therefore, further deepening on cellular and molecular impairment of patients’ immune system and its interplay with myeloma cells will help to elucidate its implication in disease progression. It would be important to ascertain whether alterations in the immune system are responsible for the progression of MGUS to MM or if in contrast, abnormalities in the tumor plasma cells induce an immunosuppressive microenvironment, favoring the transition to the malignant stages of the disease. In addition, it would be of interest to investigate whether such immune impairment is the result of a sequential additive process or otherwise consequence of a specific global alteration already present at the MGUS phase. Moreover, the precise understanding of the nature of immune alterations could contribute to the discovery of new targets for therapeutic approaches.

Additionally, it is of utmost importance to discern the immune-stimulating mechanisms of the drugs described above in the context of MM, principally of those for which controversial data have been reported as HDACi, and of the most novel ones (i.e., arginase inhibitors, IDO inhibitors, etc.). In line with this, new preclinical studies and clinical trials currently ongoing with some of them will help to elucidate their real perspectives as antimyeloma agents, and especially their capacity to potentiate the efficacy of immunotherapeutic mAbs.

## Figures and Tables

**Table 1:** Summary of the main immunomodulating effects of immunomodulatory drugs (IMiDs), proteasome inhibitors, histone deacetylase inhibitors, cyclophosphamide, arginase inhibitors and IDO inhibitors.

Drug Group	Drug Name	Target Cell Population	Immune Effects/Molecular Mechanisms	Reference
Immunomodulatory drugs (IMiDs)	Thalidomide	T cells	↑ cytotoxic responses	[155]
			↑ proliferative responses ↑ IL-2 and IFN-γ	[155,156]
		NK cells	↑ cytotoxic activity	[156]
	Lenalidomide	T cells	↑ IL-2 production	[157,158]
			↑ AP-1 transcriptional activity	[157]
			↑ activation; ↓ Ikaros and Aiolos	[159]
		NK cells	↑ cytotoxic activity	[158]
		PBMCs	↑ cytotoxic activity	[159,160]
			↑ ADCC	[158]
		MM cells	↑ MICA and PVR expression	[161]
			↓ PD-L1 expression	[35,162]
			No change in PD-L1 expression	[163]
		DCs	↑ endocytic activity ↑ MHC Class I and CD86 expression	[164]
		Treg	↓ proliferation	[165]
			↓ differentiation	[166]
	Pomalidomide	T cells	↑ IL-2 production	[157,158]
			↑ AP-1 transcriptional activity	[157]
			Nuclear translocation of AP-1 and NFAT2	[158]
		NK cells	↑ granzyme-B expression	[167]
			↑ cytotoxic activity	[158,167]
			↑ Zap-70 phosphorylation	[167]
		PBMCs	↑ ADCC	[158]
		MM cells	↑ MICA and PVR expression	[161]
			↑ CD38 expression	[168]
		DCs	↑ endocytic activity ↑ MHC Class I and CD86 expression	[164]
		Treg	↓ proliferation	[165]
Proteasome inhibitors(PIs)	Bortezomib	Tumor cells	↑ Hsp60 and Hsp90 exposure (↑ ICD induction)	[169,170]
		MM cells	↑ CALR exposure (↑ ICD induction)	[171]
	Carfilzomib	MM cells	↑ CALR exposure (↑ ICD induction)	[171]
Histone deacetylase inhibitors (HDACi)	MGCD0103 (mocetinostat)	MM cells	↑ susceptibility to lysis by MAGE-A3-specific CTLs	[172]
	Valproic acid (VPA)	MM cells	↑ MICA/B and ULBP2 expression	[173]
	Sodium butyrate	MM cells	↑ MICA expression	[174]
	Panobinostat	MM cells	↑ ULPBP2/5/6 and MICA/B expression	[163]
			↑ PD-L1 expression	[163]
			↑ CD38 expression	[175]
		DCs	Impairment of DCs function to stimulate antigen-specific immune responses	[176]
	ACY-241	DCs	↓ PD-L1 expression (pDCs) ↑ CD80, CD86 and MHC molecules (Class I and II) expression	[177,178]
		MM cells	↓ PD-L1 expression ↑ CD80, CD86 and MHC molecules (Class I and II) expression	[178]
			↑ CD38 expression	[179]
		Treg	↓ PD-L1 expression	[178]
		T cells	↓ PD-1 expression	[178]
	Entinostat	MM cells	↑ PD-L1 expression	[163]
			↑ CD38 expression	[180]
	Ricolinostat	MM cells	↑ PD-L1 expression	[163]
			↑ CD38 expression	[179]
	WT-161	MM cells	↑ CD38 expression	[179]
Alkylating agents	Cyclophosphamide	Tumor cells	↑ CALR translocation	[181,182]
			↑ release of HMGB1	[181]
		Tregs	Depletion of this population	[182,183]
		T cells	Promotes Th1 polarization	[183]
		NK cells	↑ activation	[182]
		Myeloid cells	Modulation of this population	[182]
		MM cells	↑ secretory response, ↓ CD47	[184]
		Macrophages	↑ CD64 expression, ↑ ADCP	[184]
Arginase inhibitors	nor-NOHA	High-density neutrophils	Reversion of the immune-suppressive properties of this population	[103]
	CB-1158	High-density neutrophils	Reversion of the immune-suppressive properties of this population	[103]
	BEC	M2-type macrophages	Reversion of the anti-myeloma effect of M2-type macrophages in the context of Th2 adoptive cell therapy	[185]
		MDSCs	Reversion of the inhibitory effect of conditioned media from MDSCs on the anti-myeloma efficacy of bortezomib	[102]
IDO inhibitors	INCB014943	DCs	Reversion of effector T cell suppression induced by DCs	[74]
	1-methyl-DL-Trp	IDO^+^ MM cells	Reversion of Treg expansion induced by IDO	[186]
		Osteoclasts	Reversion of the suppression of T-cell proliferation and CTLs activity induced by osteoclasts	[187]
		Macrophages	Reversion of the inhibition of CD4^+^ T cell proliferation and cytokine production induced by macrophages	[188]

## References

[1] Rajkumar S.V., Dimopoulos M.A., Palumbo A., Blade J., Merlini G., Mateos M.-V., Kumar S., Hillengass J., Kastritis E., Richardson P. (2014). International Myeloma Working Group Updated Criteria for the Diagnosis of Multiple Myeloma. Lancet Oncol..

[2] Mogollón P., Díaz-Tejedor A., Algarín E.M., Paíno T., Garayoa M., Ocio E.M. (2019). Biological Background of Resistance to Current Standards of Care in Multiple Myeloma. Cells.

[3] Garcia-Gomez A., Sanchez-Guijo F., del Cañizo M.C., San Miguel J.F., Garayoa M. (2014). Multiple Myeloma Mesenchymal Stromal Cells: Contribution to Myeloma Bone Disease and Therapeutics. World J. Stem Cells.

[4] Holstein S.A., McCarthy P.L. (2017). Immunomodulatory Drugs in Multiple Myeloma: Mechanisms of Action and Clinical Experience. Drugs.

[5] Binsfeld M., Fostier K., Muller J., Baron F., Schots R., Beguin Y., Heusschen R., Caers J. (2014). Cellular Immunotherapy in Multiple Myeloma: Lessons from Preclinical Models. Biochim. Biophys. Acta.

[6] Gonzalez H., Hagerling C., Werb Z. (2018). Roles of the Immune System in Cancer: From Tumor Initiation to Metastatic Progression. Genes Dev..

[7] Gu Y., Jin Y., Ding J., Yujie W., Shi Q., Qu X., Zhao S., Li J., Lijuan C. (2020). Low Absolute CD4þ T Cell Counts in Peripheral Blood Predict Poor Prognosis in Patients with Newly Diagnosed Multiple Myeloma. Leuk. Lymphoma.

[8] Puig N., Paíno T., Pérez J.J., Rodero E., Paiva B., Cedena M.T., Díaz-Tejedor A., Aires-Mejia I., Contreras T., Pessoa de Magalhães R. (2019). Dissecting the Bone Marrow Immune Microenvironment in the Complete Spectrum of Monoclonal Gammopathies: Potential Implications in Disease Pathogenesis. EHA Libr..

[9] Murakami H., Ogawara H., Hiroshi H. (2004). Th1/Th2 Cells in Patients with Multiple Myeloma. Hematology.

[10] Sharma A., Khan R., Joshi S., Kumar L., Sharma M. (2010). Dysregulation in T Helper 1/T Helper 2 Cytokine Ratios in Patients with Multiple Myeloma. Leuk. Lymphoma.

[11] Ogawara H., Handa H., Yamazaki T., Toda T., Yoshida K., Nishimoto N., Al-ma’Quol W.H.S., Kaneko Y., Matsushima T., Tsukamoto N. (2005). High Th1/Th2 Ratio in Patients with Multiple Myeloma. Leuk. Res..

[12] Pérez-Andres M., Almeida J., Martin-Ayuso M., Moro M.J., Martin-Nuñez G., Galende J., Hernandez J., Mateo G., San Miguel J.F., Orfao A. (2006). Characterization of Bone Marrow T Cells in Monoclonal Gammopathy of Undetermined Significance, Multiple Myeloma, and Plasma Cell Leukemia Demonstrates Increased Infiltration by Cytotoxic/Th1 T Cells Demonstrating a Squed TCR-Vbeta Repertoire. Cancer.

[13] Zhou L., Ivanov I.I., Spolski R., Min R., Shenderov K., Egawa T., Levy D.E., Leonard W.J., Littman D.R. (2007). IL-6 Programs T H-17 Cell Differentiation by Promoting Sequential Engagement of the IL-21 and IL-23 Pathways. Nat. Immunol..

[14] Prabhala R.H., Pelluru D., Fulciniti M., Prabhala H.K., Nanjappa P., Song W., Pai C., Amin S., Tai Y.-T., Richardson P.G. (2010). Elevated IL-17 Produced by TH17 Cells Promotes Myeloma Cell Growth and Inhibits Immune Function in Multiple Myeloma. Blood.

[15] Pessoa de Magalhães R.J., Vidriales M.-B., Paiva B., Fernandez-Gimenez C., García-Sanz R., Mateos M.-V., Gutierrez N.C., Lecrevisse Q., Blanco J.F., Hernández J. (2013). Analysis of the Immune System of Multiple Myeloma Patients Achieving Long-Term Disease Control by Multidimensional Flow Cytometry. Haematologica.

[16] Zavidij O., Haradhvala N.J., Mouhieddine T.H., Sklavenitis-Pistofidis R., Cai S., Reidy M., Rahmat M., Flaifel A., Ferland B., Su N.K. (2020). Single-Cell RNA Sequencing Reveals Compromised Immune Microenvironment in Precursor Stages of Multiple Myeloma. Nat. Cancer.

[17] Maecker B., Anderson K.S., Von Bergwelt-Baildon M.S., Weller E., Vonderheide R.H., Richardson P.G., Schlossman R.L., Menezes I.A., Xia Z., Munshi N.C. (2003). Viral Antigen-Specific CD8^+^ T-Cell Responses Are Impaired in Multiple Myeloma: Virus-Specific T Cells in Multiple Myeloma. Br. J. Haematol..

[18] Rossi M., Botta C., Correale P., Tassone P., Tagliaferri P. (2013). Immunologic Microenvironment and Personalized Treatment in Multiple Myeloma. Expert Opin. Biol. Ther..

[19] Campbell J.D., Cook G., Robertson S.E., Fraser A., Boyd K.S., Gracie J.A., Franklin I.M. (2001). Suppression of IL-2-Induced T Cell Proliferation and Phosphorylation of STAT3 and STAT5 by Tumor-Derived TGF Beta Is Reversed by IL-15. J. Immunol..

[20] Gorelik L., Flavell R.A. (2002). Transforming Growth Factor-Beta in T-Cell Biology. Nat. Rev. Immunol..

[21] Quarona V., Ferri V., Chillemi A., Bolzoni M., Mancini C., Zaccarello G., Roato I., Morandi F., Marimpietri D., Faccani G. (2015). Unraveling the Contribution of Ectoenzymes to Myeloma Life and Survival in the Bone Marrow Niche: Ectoenzymes and the Myeloma Niche. Ann. N. Y. Acad. Sci..

[22] Malavasi F., Deaglio S., Funaro A., Ferrero E., Horenstein A.L., Ortolan E., Vaisitti T., Aydin S. (2008). Evolution and Function of the ADP Ribosyl Cyclase/CD38 Gene Family in Physiology and Pathology. Physiol. Rev..

[23] Antonioli L., Blandizzi C., Pacher P., Haskó G. (2013). Immunity, Inflammation and Cancer: A Leading Role for Adenosine. Nat. Rev. Cancer.

[24] Greenwald R.J., Freeman G.J., Sharpe A.H. (2005). The B7 Family Revisited. Annu. Rev. Immunol..

[25] Pardoll D.M. (2012). The Blockade of Immune Checkpoints in Cancer Immunotherapy. Nat. Rev. Cancer.

[26] Parry R.V., Chemnitz J.M., Frauwirth K.A., Lanfranco A.R., Braunstein I., Kobayashi S.V., Linsley P.S., Thompson C.B., Riley J.L. (2005). CTLA-4 and PD-1 Receptors Inhibit T-Cell Activation by Distinct Mechanisms. Mol. Cell. Biol..

[27] Riley J.L. (2009). PD-1 Signaling in Primary T Cells. Immunol. Rev..

[28] Sharpe A.H., Pauken K.E. (2018). The Diverse Functions of the PD1 Inhibitory Pathway. Nat. Rev. Immunol..

[29] Liu J., Hamrouni A., Wolowiec D., Coiteux V., Kuliczkowski K., Hetuin D., Saudemont A., Quesnel B. (2007). Plasma Cells from Multiple Myeloma Patients Express B7-H1 (PD-L1) and Increase Expression after Stimulation with IFN-γ and TLR Ligands via a MyD88-, TRAF6-, and MEK-Dependent Pathway. Blood.

[30] Paiva B., Azpilikueta A., Puig N., Ocio E.M., Sharma R., Oyajobi B.O., Labiano S., San-Segundo L., Rodriguez A., Aires-Mejia I. (2015). PD-L1/PD-1 Presence in the Tumor Microenvironment and Activity of PD-1 Blockade in Multiple Myeloma. Leukemia.

[31] Tamura H., Ishibashi M., Yamashita T., Tanosaki S., Okuyama N., Kondo A., Hyodo H., Shinya E., Takahashi H., Dong H. (2013). Marrow Stromal Cells Induce B7-H1 Expression on Myeloma Cells, Generating Aggressive Characteristics in Multiple Myeloma. Leukemia.

[32] Yousef S., Marvin J., Steinbach M., Langemo A., Kovacsovics T., Binder M., Kröger N., Luetkens T., Atanackovic D. (2015). Immunomodulatory Molecule PD-L1 Is Expressed on Malignant Plasma Cells and Myeloma-Propagating Pre-Plasma Cells in the Bone Marrow of Multiple Myeloma Patients. Blood Cancer J..

[33] Tamura H., Ishibashi M., Sunakawa-Kii M., Inokuchi K. (2020). PD-L1–PD-1 Pathway in the Pathophysiology of Multiple Myeloma. Cancers.

[34] Rosenblatt J., Glotzbecker B., Mills H., Vasir B., Tzachanis D., Levine J.D., Joyce R.M., Wellenstein K., Keefe W., Schickler M. (2011). PD-1 Blockade by CT-011, Anti PD-1 Antibody, Enhances Ex-Vivo T Cell Responses to Autologous Dendritic/Myeloma Fusion Vaccine. J. Immunother..

[35] Benson D.M., Bakan C.E., Mishra A., Hofmeister C.C., Efebera Y., Becknell B., Baiocchi R.A., Zhang J., Yu J., Smith M.K. (2010). The PD-1/PD-L1 Axis Modulates the Natural Killer Cell versus Multiple Myeloma Effect: A Therapeutic Target for CT-011, a Novel Monoclonal Anti–PD-1 Antibody. Blood.

[36] Frigola X., Inman B.A., Lohse C.M., Krco C.J., Cheville J.C., Thompson R.H., Leibovich B., Blute M.L., Dong H., Kwon E.D. (2011). Identification of a Soluble Form of B7-H1 That Retains Immunosuppressive Activity and Is Associated with Aggressive Renal Cell Carcinoma. Clin. Cancer Res..

[37] Wang L., Wang H., Chen H., Wang W., Chen X.-Q., Geng Q.-R., Xia Z.-J., Lu Y. (2015). Serum Levels of Soluble Programmed Death Ligand 1 Predict Treatment Response and Progression Free Survival in Multiple Myeloma. Oncotarget.

[38] Sunakawa M., Tamura H., Ishibashi M., Sasaki M., Handa H., Imai Y., Tanaka J., Tanosaki S., Ito S., Inokuchi K. (2017). Clinical Impact and Possible Immunosuppressive Function of Soluble B7-H1 (PD-L1) in Multiple Myeloma. Clin. Lymphoma Myeloma Leuk..

[39] Ishibashi M., Tamura H., Sunakawa M., Kondo-Onodera A., Okuyama N., Hamada Y., Moriya K., Choi I., Tamada K., Inokuchi K. (2016). Myeloma Drug Resistance Induced by Binding of Myeloma B7-H1 (PD-L1) to PD-1. Cancer Immunol. Res..

[40] Hallett W.H.D., Jing W., Drobyski W.R., Johnson B.D. (2011). Immunosuppressive Effects of Multiple Myeloma Are Overcome by PD-L1 Blockade. Biol. Blood Marrow. Transplant..

[41] Puig N., Corchete-Sánchez L.A., Pérez-Morán J.J., Dávila J., Paíno T., de la Rubia J., Oriol A., Martín-Sánchez J., de Arriba F., Bladé J. (2020). Pembrolizumab as Consolidation Strategy in Patients with Multiple Myeloma: Results of the GEM-Pembresid Clinical Trial. Cancers.

[42] Badros A., Hyjek E., Ma N., Lesokhin A., Dogan A., Rapoport A.P., Kocoglu M., Lederer E., Philip S., Milliron T. (2017). Pembrolizumab, Pomalidomide, and Low-Dose Dexamethasone for Relapsed/Refractory Multiple Myeloma. Blood.

[43] Mateos M.-V., Orlowski R.Z., Ocio E.M., Rodríguez-Otero P., Reece D., Moreau P., Munshi N., Avigan D.E., Siegel D.S., Ghori R. (2019). Pembrolizumab Combined with Lenalidomide and Low-Dose Dexamethasone for Relapsed or Refractory Multiple Myeloma: Phase I KEYNOTE-023 Study. Br. J. Haematol..

[44] Mateos M.-V., Blacklock H., Schjesvold F., Oriol A., Simpson D., George A., Goldschmidt H., Larocca A., Chanan-Khan A., Sherbenou D. (2019). Pembrolizumab plus Pomalidomide and Dexamethasone for Patients with Relapsed or Refractory Multiple Myeloma (KEYNOTE-183): A Randomised, Open-Label, Phase 3 Trial. Lancet Haematol..

[45] Usmani S.Z., Schjesvold F., Oriol A., Karlin L., Cavo M., Rifkin R.M., Yimer H.A., LeBlanc R., Takezako N., McCroskey R.D. (2019). Pembrolizumab plus Lenalidomide and Dexamethasone for Patients with Treatment-Naive Multiple Myeloma (KEYNOTE-185): A Randomised, Open-Label, Phase 3 Trial. Lancet Haematol..

[46] Alegre M.L., Frauwirth K.A., Thompson C.B. (2001). T-Cell Regulation by CD28 and CTLA-4. Nat. Rev. Immunol..

[47] Skarbnik A.P., Donato M.L., Korngold R., Feinman R., Rowley S.D., Goy A., Vesole D.H., Munshi P.N., Siegel D.S., Feldman T.A. (2018). Safety and Efficacy Data for Combined Checkpoint Inhibition with Ipilimumab (Ipi) and Nivolumab (Nivo) As Consolidation Following Autologous Stem Cell Transplantation (ASCT) for High-Risk Hematological Malignancies—CPIT-001 Trial. Blood.

[48] Vivier E., Raulet D.H., Moretta A., Caligiuri M.A., Zitvogel L., Lanier L.L., Yokoyama W.M., Ugolini S. (2011). Innate or Adaptive Immunity? The Example of Natural Killer Cells. Science.

[49] Morvan M.G., Lanier L.L. (2016). NK Cells and Cancer: You Can Teach Innate Cells New Tricks. Nat. Rev. Cancer.

[50] Carbone E., Neri P., Mesuraca M., Fulciniti M.T., Otsuki T., Pende D., Groh V., Spies T., Pollio G., Cosman D. (2005). HLA Class I, NKG2D, and Natural Cytotoxicity Receptors Regulate Multiple Myeloma Cell Recognition by Natural Killer Cells. Blood.

[51] Jinushi M., Vanneman M., Munshi N.C., Tai Y.-T., Prabhala R.H., Ritz J., Neuberg D., Anderson K.C., Carrasco D.R., Dranoff G. (2008). MHC Class I Chain-Related Protein A Antibodies and Shedding Are Associated with the Progression of Multiple Myeloma. Proc. Natl. Acad. USA.

[52] von Lilienfeld-Toal M., Frank S., Leyendecker C., Feyler S., Jarmin S., Morgan R., Glasmacher A., Märten A., Schmidt-Wolf I.G.H., Brossart P. (2010). Reduced Immune Effector Cell NKG2D Expression and Increased Levels of Soluble NKG2D Ligands in Multiple Myeloma May Not Be Causally Linked. Cancer Immunol. Immunother..

[53] Bailur J.K., McCachren S.S., Doxie D.B., Shrestha M., Pendleton K., Nooka A.K., Neparidze N., Parker T.L., Bar N., Kaufman J.L. (2019). Early Alterations in Stem-like/Marrow-Resident T Cells and Innate and Myeloid Cells in Preneoplastic Gammopathy. JCI Insight..

[54] Fauriat C., Mallet F., Olive D., Costello R.T. (2006). Impaired Activating Receptor Expression Pattern in Natural Killer Cells from Patients with Multiple Myeloma. Leukemia.

[55] El-Sherbiny Y.M., Meade J.L., Holmes T.D., McGonagle D., Mackie S.L., Morgan A.W., Cook G., Feyler S., Richards S.J., Davies F.E. (2007). The Requirement for DNAM-1, NKG2D, and NKp46 in the Natural Killer Cell-Mediated Killing of Myeloma Cells. Cancer Res..

[56] Allan D.S.J., Rybalov B., Awong G., Zúñiga-Pflücker J.C., Kopcow H.D., Carlyle J.R., Strominger J.L. (2010). TGF-β Affects Development and Differentiation of Human Natural Killer Cell Subsets. Eur. J. Immunol..

[57] Vulpis E., Cecere F., Molfetta R., Soriani A., Fionda C., Peruzzi G., Caracciolo G., Palchetti S., Masuelli L., Simonelli L. (2017). Genotoxic Stress Modulates the Release of Exosomes from Multiple Myeloma Cells Capable of Activating NK Cell Cytokine Production: Role of HSP70/TLR2/NF-KB Axis. OncoImmunology.

[58] Borrelli C., Ricci B., Vulpis E., Fionda C., Ricciardi M.R., Petrucci M.T., Masuelli L., Peri A., Cippitelli M., Zingoni A. (2018). Drug-Induced Senescent Multiple Myeloma Cells Elicit NK Cell Proliferation by Direct or Exosome-Mediated IL15 *Trans.* -Presentation. Cancer Immunol. Res..

[59] Baginska J., Viry E., Paggetti J., Medves S., Berchem G., Moussay E., Janji B. (2013). The Critical Role of the Tumor Microenvironment in Shaping Natural Killer Cell-Mediated Anti-Tumor Immunity. Front. Immunol..

[60] Garg T.K., Gann J.I., Malaviarachchi P.A., Stone K., Macleod V., Greenway A.D., Akel N.S., Edmondson R.D., Davies F.E., Epstein J. (2016). Myeloma-Derived Exosomes and Soluble Factors Suppress Natural Killer Cell Function. Blood.

[61] Cooper M.D. (2015). The Early History of B Cells. Nat. Rev. Immunol..

[62] Pilarski L.M., Andrews E.J., Mant M.J., Ruether B.A. (1986). Humoral Immune Deficiency in Multiple Myeloma Patients Due to Compromised B-Cell Function. J. Clin. Immunol..

[63] Rawstron A.C., Davies F.E., Owen R.G., English A., Pratt G., Child J.A., Jack A.S., Morgan G.J. (1998). B-Lymphocyte Suppression in Multiple Myeloma Is a Reversible Phenomenon Specific to Normal B-Cell Progenitors and Plasma Cell Precursors. Br. J. Haematol..

[64] Kyle R.A., Remstein E.D., Therneau T.M., Dispenzieri A., Kurtin P.J., Hodnefield J.M., Larson D.R., Plevak M.F., Jelinek D.F., Fonseca R. (2007). Clinical Course and Prognosis of Smoldering (Asymptomatic) Multiple Myeloma. N. Engl. J. Med..

[65] Zou Z., Guo T., Cui J., Zhang L., Pan L. (2019). Onset of Regulatory B Cells Occurs at Initial Stage of B Cell Dysfunction in Multiple Myeloma. Blood.

[66] Zhang L., Tai Y.-T., Ho M., Xing L., Chauhan D., Gang A., Qiu L., Anderson K.C. (2017). Regulatory B Cell-Myeloma Cell Interaction Confers Immunosuppression and Promotes Their Survival in the Bone Marrow Milieu. Blood Cancer J..

[67] Banchereau J., Briere F., Caux C., Davoust J., Lebecque S., Liu Y.-J., Pulendran B., Palucka K. (2000). Immunobiology of Dendritic Cells. Annu. Rev. Immunol..

[68] Chen P., Denniston A.K., Hirani S., Hannes S., Nussenblatt R.B. (2015). Role of Dendritic Cell Subsets in Immunity and Their Contribution to Non-Infectious Uveitis. Surv. Ophthalmol..

[69] Raje N., Gong J., Chauhan D., Teoh G., Avigan D., Wu Z., Chen D., Treon S.P., Webb I.J., Kufe D.W. (1999). Bone Marrow and Peripheral Blood Dendritic Cells From Patients with Multiple Myeloma Are Phenotypically and Functionally Normal Despite the Detection of Kaposi’s Sarcoma Herpesvirus Gene Sequences. Blood.

[70] Brown R.D., Pope B., Murray A., Esdale W., Sze D.M., Gibson J., Ho P.J., Hart D., Joshua D. (2001). Dendritic Cells from Patients with Myeloma Are Numerically Normal but Functionally Defective as They Fail to Up-Regulate CD80 (B7-1) Expression after HuCD40LT Stimulation Because of Inhibition by Transforming Growth Factor-Beta1 and Interleukin-10. Blood.

[71] Ratta M., Fagnoni F., Curti A., Vescovini R., Sansoni P., Oliviero B., Fogli M., Ferri E., Cuna G.R.D., Tura S. (2002). Dendritic Cells Are Functionally Defective in Multiple Myeloma: The Role of Interleukin-6. Blood.

[72] Brimnes M.K., Svane I.M., Johnsen H.E. (2006). Impaired Functionality and Phenotypic Profile of Dendritic Cells from Patients with Multiple Myeloma. Clin. Exp. Immunol..

[73] Leone P., Berardi S., Frassanito M.A., Ria R., De Re V., Cicco S., Battaglia S., Ditonno P., Dammacco F., Vacca A. (2015). Dendritic Cells Accumulate in the Bone Marrow of Myeloma Patients Where They Protect Tumor Plasma Cells from CD8+ T-Cell Killing. Blood.

[74] Nair J.R., Carlson L.M., Koorella C., Rozanski C.H., Byrne G.E., Bergsagel P.L., Shaughnessy J.P., Boise L.H., Chanan-Khan A., Lee K.P. (2011). CD28 Expressed on Malignant Plasma Cells Induces a Prosurvival and Immunosuppressive Microenvironment. J. Immunol..

[75] Platten M., Wick W., Van den Eynde B.J. (2012). Tryptophan Catabolism in Cancer: Beyond IDO and Tryptophan Depletion. Cancer Res..

[76] Munn D.H., Sharma M.D., Baban B., Harding H.P., Zhang Y., Ron D., Mellor A.L. (2005). GCN2 Kinase in T Cells Mediates Proliferative Arrest and Anergy Induction in Response to Indoleamine 2,3-Dioxygenase. Immunity.

[77] Munn D.H., Mellor A.L. (2016). IDO in the Tumor Microenvironment: Inflammation, Counter-Regulation and Tolerance. Trends Immunol..

[78] Sponaas A.-M., Moharrami N.N., Feyzi E., Standal T., Rustad E.H., Waage A., Sundan A. (2015). PDL1 Expression on Plasma and Dendritic Cells in Myeloma Bone Marrow Suggests Benefit of Targeted Anti PD1-PDL1 Therapy. PLoS ONE.

[79] Strobl H., Knapp W. (1999). TGF-Beta1 Regulation of Dendritic Cells. Microbes Infect..

[80] Zheng Y., Cai Z., Wang S., Zhang X., Qian J., Hong S., Li H., Wang M., Yang J., Yi Q. (2009). Macrophages Are an Abundant Component of Myeloma Microenvironment and Protect Myeloma Cells from Chemotherapy Drug-Induced Apoptosis. Blood.

[81] Kim J., Denu R.A., Dollar B.A., Escalante L.E., Kuether J.P., Callander N.S., Asimakopoulos F., Hematti P. (2012). Macrophages and Mesenchymal Stromal Cells Support Survival and Proliferation of Multiple Myeloma Cells. Br. J. Haematol..

[82] Berardi S., Ria R., Reale A., De Luisi A., Catacchio I., Moschetta M., Vacca A. (2013). Multiple Myeloma Macrophages: Pivotal Players in the Tumor Microenvironment. J. Oncol..

[83] Suyanı E., Sucak G.T., Akyürek N., Şahin S., Baysal N.A., Yağcı M., Haznedar R. (2013). Tumor-Associated Macrophages as a Prognostic Parameter in Multiple Myeloma. Ann. Hematol..

[84] Andersen M.N., Andersen N.F., Rødgaard-Hansen S., Hokland M., Abildgaard N., Møller H.J. (2015). The Novel Biomarker of Alternative Macrophage Activation, Soluble Mannose Receptor (SMR/SCD206): Implications in Multiple Myeloma. Leuk. Res..

[85] Panchabhai S., Kelemen K., Ahmann G., Sebastian S., Mantei J., Fonseca R. (2016). Tumor-Associated Macrophages and Extracellular Matrix Metalloproteinase Inducer in Prognosis of Multiple Myeloma. Leukemia.

[86] Beyar-Katz O., Magidey K., Reiner-Benaim A., Barak N., Avivi I., Cohen Y., Timaner M., Avraham S., Hayun M., Lavi N. (2019). Proinflammatory Macrophages Promote Multiple Myeloma Resistance to Bortezomib Therapy. Mol. Cancer Res..

[87] Beider K., Bitner H., Leiba M., Gutwein O., Koren-Michowitz M., Ostrovsky O., Abraham M., Wald H., Galun E., Peled A. (2014). Multiple Myeloma Cells Recruit Tumor-Supportive Macrophages through the CXCR4/CXCL12 Axis and Promote Their Polarization toward the M2 Phenotype. Oncotarget.

[88] Cassetta L., Baekkevold E.S., Brandau S., Bujko A., Cassatella M.A., Dorhoi A., Krieg C., Lin A., Loré K., Marini O. (2019). Deciphering Myeloid-Derived Suppressor Cells: Isolation and Markers in Humans, Mice and Non-Human Primates. Cancer Immunol. Immunother..

[89] Ramachandran I.R., Martner A., Pisklakova A., Condamine T., Chase T., Vogl T., Roth J., Gabrilovich D., Nefedova Y. (2013). Myeloid-Derived Suppressor Cells Regulate Growth of Multiple Myeloma by Inhibiting T Cells in Bone Marrow. J. Immunol..

[90] Favaloro J., Liyadipitiya T., Brown R., Yang S., Suen H., Woodland N., Nassif N., Hart D., Fromm P., Weatherburn C. (2014). Myeloid Derived Suppressor Cells Are Numerically, Functionally and Phenotypically Different in Patients with Multiple Myeloma. Leuk. Lymphoma.

[91] Giallongo C., Tibullo D., Parrinello N.L., La Cava P., Di Rosa M., Bramanti V., Di Raimondo C., Conticello C., Chiarenza A., Palumbo G.A. (2016). Granulocyte-like Myeloid Derived Suppressor Cells (G-MDSC) Are Increased in Multiple Myeloma and Are Driven by Dysfunctional Mesenchymal Stem Cells (MSC). Oncotarget.

[92] Görgün G.T., Whitehill G., Anderson J.L., Hideshima T., Maguire C., Laubach J., Raje N., Munshi N.C., Richardson P.G., Anderson K.C. (2013). Tumor-Promoting Immune-Suppressive Myeloid-Derived Suppressor Cells in the Multiple Myeloma Microenvironment in Humans. Blood.

[93] Malek E., de Lima M., Letterio J.J., Kim B.-G., Finke J.H., Driscoll J.J., Giralt S.A. (2016). Myeloid-Derived Suppressor Cells: The Green Light for Myeloma Immune Escape. Blood Rev..

[94] Sawant A., Ponnazhagan S. (2013). Myeloid-Derived Suppressor Cells as Osteoclast Progenitors: A Novel Target for Controlling Osteolytic Bone Metastasis. Cancer Res..

[95] Veirman K.D., Ginderachter J.A.V., Lub S., Beule N.D., Thielemans K., Bautmans I., Oyajobi B.O. (2015). Multiple Myeloma Induces Mcl-1 Expression and Survival of Myeloid-Derived Suppressor Cells. Oncotarget.

[96] Yu H., Pardoll D., Jove R. (2009). STATs in Cancer Inflammation and Immunity: A Leading Role for STAT3. Nat. Rev. Cancer.

[97] Budhwar S., Verma P., Verma R., Rai S., Singh K. (2018). The Yin and Yang of Myeloid Derived Suppressor Cells. Front. Immunol..

[98] Waldron T.J., Quatromoni J.G., Karakasheva T.A., Singhal S., Rustgi A.K. (2013). Myeloid Derived Suppressor Cells. Oncoimmunology.

[99] Wang J., De Veirman K., De Beule N., Maes K., De Bruyne E., Van Valckenborgh E., Vanderkerken K., Menu E. (2015). The Bone Marrow Microenvironment Enhances Multiple Myeloma Progression by Exosome-Mediated Activation of Myeloid-Derived Suppressor Cells. Oncotarget.

[100] Wang J., Veirman K.D., Faict S., Frassanito M.A., Ribatti D., Vacca A., Menu E. (2016). Multiple Myeloma Exosomes Establish a Favourable Bone Marrow Microenvironment with Enhanced Angiogenesis and Immunosuppression. J. Pathol.

[101] Mayadas T.N., Cullere X., Lowell C.A. (2014). The Multifaceted Functions of Neutrophils. Annu. Rev. Pathol. Mech. Dis..

[102] Romano A., Parrinello N.L., La Cava P., Tibullo D., Giallongo C., Camiolo G., Puglisi F., Parisi M., Pirosa M.C., Martino E. (2018). PMN-MDSC and Arginase Are Increased in Myeloma and May Contribute to Resistance to Therapy. Expert Rev. Mol. Diagn..

[103] Romano A., Parrinello N.L., Simeon V., Puglisi F., La Cava P., Bellofiore C., Giallongo C., Camiolo G., D’Auria F., Grieco V. (2020). High-Density Neutrophils in MGUS and Multiple Myeloma Are Dysfunctional and Immune-Suppressive Due to Increased STAT3 Downstream Signaling. Sci. Rep..

[104] Monu N.R., Frey A.B. (2012). Myeloid-Derived Suppressor Cells and Anti-Tumor T Cells: A Complex Relationship. Immunol. Investig..

[105] Puglisi F., Parrinello N.L., Giallongo C., Cambria D., Camiolo G., Bellofiore C., Conticello C., Del Fabro V., Leotta V., Markovic U. (2019). Plasticity of High-Density Neutrophils in Multiple Myeloma Is Associated with Increased Autophagy Via STAT3. Int. J. Mol. Sci..

[106] Romano A., Laura Parrinello N., Cerchione C., Letizia Consoli M., Parisi M., Calafiore V., Martino E., Conticello C., Di Raimondo F., Alberto Palumbo G. (2017). The NLR and LMR Ratio in Newly Diagnosed MM Patients Treated Upfront with Novel Agents. Blood Cancer J..

[107] Solmaz Medeni S., Acar C., Olgun A., Acar A., Seyhanlı A., Taskıran E., Sevindik O.G., Alacacıoglu I., Piskin O., Ozcan M.A. (2018). Can Neutrophil-to-Lymphocyte Ratio, Monocyte-to-Lymphocyte Ratio, and Platelet-to-Lymphocyte Ratio at Day +100 Be Used as a Prognostic Marker in Multiple Myeloma Patients with Autologous Transplantation?. Clin. Transplant..

[108] Alrasheed N., Lee L., Ghorani E., Henry J.Y., Conde L., Chin M., Galas-Filipowicz D., Furness A.J.S., Chavda S.J., Richards H. (2020). Marrow-Infiltrating Regulatory T Cells Correlate with the Presence of Dysfunctional CD4+PD-1+ Cells and Inferior Survival in Patients with Newly Diagnosed Multiple Myeloma. Clin. Cancer Res..

[109] Ohue Y., Nishikawa H. (2019). Regulatory T (Treg) Cells in Cancer: Can Treg Cells Be a New Therapeutic Target?. Cancer Sci..

[110] Grohmann U., Orabona C., Fallarino F., Vacca C., Calcinaro F., Falorni A., Candeloro P., Belladonna M.L., Bianchi R., Fioretti M.C. (2002). CTLA-4-Ig Regulates Tryptophan Catabolism in Vivo. Nat. Immunol..

[111] Braga W.M.T., da Silva B.R., de Carvalho A.C., Maekawa Y.H., Bortoluzzo A.B., Rizzatti E.G., Atanackovic D., Colleoni G.W.B. (2014). FOXP3 and CTLA4 Overexpression in Multiple Myeloma Bone Marrow as a Sign of Accumulation of CD4(+) T Regulatory Cells. Cancer Immunol. Immunother..

[112] Muthu Raja K.R., Rihova L., Zahradova L., Klincova M., Penka M., Hajek R. (2012). Increased T Regulatory Cells Are Associated with Adverse Clinical Features and Predict Progression in Multiple Myeloma. PLoS ONE.

[113] Beyer M., Kochanek M., Giese T., Endl E., Weihrauch M.R., Knolle P.A., Classen S., Schultze J.L. (2006). In Vivo Peripheral Expansion of Naive CD4+CD25high FoxP3+ Regulatory T Cells in Patients with Multiple Myeloma. Blood.

[114] Feyler S., von Lilienfeld-Toal M., Jarmin S., Marles L., Rawstron A., Ashcroft A.J., Owen R.G., Selby P.J., Cook G. (2009). CD4(+)CD25(+)FoxP3(+) Regulatory T Cells Are Increased Whilst CD3(+)CD4(-)CD8(-)AlphabetaTCR(+) Double Negative T Cells Are Decreased in the Peripheral Blood of Patients with Multiple Myeloma Which Correlates with Disease Burden. Br. J. Haematol..

[115] Wang J., Cao X., Zhao A., Cai H., Wang X., Li J. (2018). Increased Activated Regulatory T Cell Subsets and Aging Treg-like Cells in Multiple Myeloma and Monoclonal Gammopathy of Undetermined Significance: A Case Control Study. Cancer Cell Int..

[116] Giannopoulos K., Kaminska W., Hus I., Dmoszynska A. (2012). The Frequency of T Regulatory Cells Modulates the Survival of Multiple Myeloma Patients: Detailed Characterisation of Immune Status in Multiple Myeloma. Br. J. Cancer.

[117] Drugs@FDA: FDA-Approved Drugs. https://www.accessdata.fda.gov/scripts/cder/daf/index.cfm?event=overview.process&varApplNo=761035.

[118] Hsi E.D., Steinle R., Balasa B., Szmania S., Draksharapu A., Shum B.P., Huseni M., Powers D., Nanisetti A., Zhang Y. (2008). CS1, a Potential New Therapeutic Antibody Target for the Treatment of Multiple Myeloma. Clin. Cancer Res..

[119] Boles K.S., Mathew P.A. (2001). Molecular Cloning of CS1, a Novel Human Natural Killer Cell Receptor Belonging to the CD2 Subset of the Immunoglobulin Superfamily. Immunogenetics.

[120] Bouchon A., Cella M., Grierson H.L., Cohen J.I., Colonna M. (2001). Cutting Edge: Activation of NK Cell-Mediated Cytotoxicity by a SAP-Independent Receptor of the CD2 Family. J. Immunol..

[121] Tai Y.-T., Dillon M., Song W., Leiba M., Li X.-F., Burger P., Lee A.I., Podar K., Hideshima T., Rice A.G. (2008). Anti-CS1 Humanized Monoclonal Antibody HuLuc63 Inhibits Myeloma Cell Adhesion and Induces Antibody-Dependent Cellular Cytotoxicity in the Bone Marrow Milieu. Blood.

[122] Collins S.M., Bakan C.E., Swartzel G.D., Hofmeister C.C., Efebera Y.A., Kwon H., Starling G.C., Ciarlariello D., Bhaskar S., Briercheck E.L. (2013). Elotuzumab Directly Enhances NK Cell Cytotoxicity against Myeloma via CS1 Ligation: Evidence for Augmented NK Cell Function Complementing ADCC. Cancer Immunol. Immunother..

[123] Balasa B., Huseni M., Cherukuri J., Steinle R., Nanisetti A., Afar D., Hsi E., Vexler V. (2008). Elotuzumab (HuLuc63) Activates CD56dim Natural Killer Cells and Monocytes Resulting in the Release of IP-10 and MCP-1. Blood.

[124] Lokhorst H.M., Plesner T., Laubach J.P., Nahi H., Gimsing P., Hansson M., Minnema M.C., Lassen U., Krejcik J., Palumbo A. (2015). Targeting CD38 with Daratumumab Monotherapy in Multiple Myeloma. N. Engl. J. Med..

[125] Lonial S., Weiss B.M., Usmani S.Z., Singhal S., Chari A., Bahlis N.J., Belch A., Krishnan A., Vescio R.A., Mateos M.V. (2016). Daratumumab Monotherapy in Patients with Treatment-Refractory Multiple Myeloma (SIRIUS): An Open-Label, Randomised, Phase 2 Trial. Lancet.

[126] Mateos M.-V., Dimopoulos M.A., Cavo M., Suzuki K., Jakubowiak A., Knop S., Doyen C., Lucio P., Nagy Z., Kaplan P. (2018). Daratumumab plus Bortezomib, Melphalan, and Prednisone for Untreated Myeloma. N. Engl. J. Med.

[127] Feng X., Zhang L., Acharya C., An G., Wen K., Qiu L., Munshi N.C., Tai Y.-T., Anderson K.C. (2017). Targeting CD38 Suppresses Induction and Function of T Regulatory Cells to Mitigate Immunosuppression in Multiple Myeloma. Clin. Cancer Res..

[128] Chillemi A. (2014). CD38 and Bone Marrow Microenvironment. Front. Biosci..

[129] Zhu C., Song Z., Wang A., Srinivasan S., Yang G., Greco R., Theilhaber J., Shehu E., Wu L., Yang Z.-Y. (2020). Isatuximab Acts through Fc-Dependent, Independent, and Direct Pathways to Kill Multiple Myeloma Cells. Front. Immunol..

[130] Ghose J., Viola D., Terrazas C., Caserta E., Troadec E., Khalife J., Gunes E.G., Sanchez J., McDonald T., Marcucci G. (2018). Daratumumab Induces CD38 Internalization and Impairs Myeloma Cell Adhesion. Oncoimmunology.

[131] Iqbal J., Zaidi M. (2006). Extracellular NAD+ Metabolism Modulates Osteoclastogenesis. Biochem. Biophys. Res. Commun..

[132] de Weers M., Tai Y.-T., van der Veer M.S., Bakker J.M., Vink T., Jacobs D.C.H., Oomen L.A., Peipp M., Valerius T., Slootstra J.W. (2011). Daratumumab, a Novel Therapeutic Human CD38 Monoclonal Antibody, Induces Killing of Multiple Myeloma and Other Hematological Tumors. J. Immunol..

[133] Overdijk M.B., Jansen J.H.M., Nederend M., Lammerts van Bueren J.J., Groen R.W.J., Parren P.W.H.I., Leusen J.H.W., Boross P. (2016). The Therapeutic CD38 Monoclonal Antibody Daratumumab Induces Programmed Cell Death via Fcγ Receptor-Mediated Cross-Linking. J. Immunol..

[134] Overdijk M.B., Verploegen S., Bögels M., van Egmond M., Lammerts van Bueren J.J., Mutis T., Groen R.W.J., Breij E., Martens A.C.M., Bleeker W.K. (2015). Antibody-Mediated Phagocytosis Contributes to the Anti-Tumor Activity of the Therapeutic Antibody Daratumumab in Lymphoma and Multiple Myeloma. MAbs.

[135] Krejcik J., Frerichs K.A., Nijhof I.S., van Kessel B., van Velzen J.F., Bloem A.C., Broekmans M.E.C., Zweegman S., van Meerloo J., Musters R.J.P. (2017). Monocytes and Granulocytes Reduce CD38 Expression Levels on Myeloma Cells in Patients Treated with Daratumumab. Clin. Cancer Res..

[136] Wang Y., Zhang Y., Hughes T., Zhang J., Caligiuri M.A., Benson D.M., Yu J. (2018). Fratricide of NK Cells in Daratumumab Therapy for Multiple Myeloma Overcome by Ex Vivo Expanded Autologous NK Cells. Clin. Cancer Res..

[137] Casneuf T., Xu X.S., Adams H.C., Axel A.E., Chiu C., Khan I., Ahmadi T., Yan X., Lonial S., Plesner T. (2017). Effects of Daratumumab on Natural Killer Cells and Impact on Clinical Outcomes in Relapsed or Refractory Multiple Myeloma. Blood Adv..

[138] Krejcik J., Casneuf T., Nijhof I.S., Verbist B., Bald J., Plesner T., Syed K., Liu K., van de Donk N.W.C.J., Weiss B.M. (2016). Daratumumab Depletes CD38+ Immune Regulatory Cells, Promotes T-Cell Expansion, and Skews T-Cell Repertoire in Multiple Myeloma. Blood.

[139] Viola D., Dona A., Caserta E., Troadec E., Besi F., McDonald T., Ghoda L., Gunes E.G., Sanchez J.F., Khalife J. (2020). Daratumumab Induces Mechanisms of Immune Activation through CD38+ NK Cell Targeting. Leukemia.

[140] Dhillon S. (2020). Isatuximab: First Approval. Drugs.

[141] Deckert J., Wetzel M.-C., Bartle L.M., Skaletskaya A., Goldmacher V.S., Vallée F., Zhou-Liu Q., Ferrari P., Pouzieux S., Lahoute C. (2014). SAR650984, a Novel Humanized CD38-Targeting Antibody, Demonstrates Potent Antitumor Activity in Models of Multiple Myeloma and Other CD38+ Hematologic Malignancies. Clin. Cancer Res..

[142] Jiang H., Acharya C., An G., Zhong M., Feng X., Wang L., Dasilva N., Song Z., Yang G., Adrian F. (2016). SAR650984 Directly Induces Multiple Myeloma Cell Death via Lysosomal-Associated and Apoptotic Pathways, Which Is Further Enhanced by Pomalidomide. Leukemia.

[143] Moreno L., Perez C., Zabaleta A., Manrique I., Alignani D., Ajona D., Blanco L., Lasa M., Maiso P., Rodriguez I. (2019). The Mechanism of Action of the Anti-CD38 Monoclonal Antibody Isatuximab in Multiple Myeloma. Clin. Cancer Res..

[144] Kennedy B.E., Sadek M., Elnenaei M.O., Reiman A., Gujar S.A. (2020). Targeting NAD+ Synthesis to Potentiate CD38-Based Immunotherapy of Multiple Myeloma. Trends Cancer.

[145] Martin T.G., Corzo K., Chiron M., van de Velde H., Abbadessa G., Campana F., Solanki M., Meng R., Lee H., Wiederschain D. (2019). Therapeutic Opportunities with Pharmacological Inhibition of CD38 with Isatuximab. Cells.

[146] Lammerts van Bueren J., Jakobs D., Kaldenhoven N., Roza M., Hiddingh S., Meesters J., Voorhorst M., Gresnigt E., Wiegman L., Ortiz Buijsse A. (2014). Direct in Vitro Comparison of Daratumumab with Surrogate Analogs of CD38 Antibodies MOR03087, SAR650984 and Ab79. Blood.

[147] Tai Y.-T., Mayes P.A., Acharya C., Zhong M.Y., Cea M., Cagnetta A., Craigen J., Yates J., Gliddon L., Fieles W. (2014). Novel Anti–B-Cell Maturation Antigen Antibody-Drug Conjugate (GSK2857916) Selectively Induces Killing of Multiple Myeloma. Blood.

[148] Montes de Oca R., Bhattacharya S., Vitali N., Patel K., Kaczynski H., Shi H.Z., Blackwell C., Seestaller-Wehr L., Cooper D., Jackson H. (2019). The Anti-BCMA Antibody-Drug Conjugate GSK2857916 Drives Immunogenic Cell Death and Immune-Mediated Anti-Tumor Responses, and in Combination with an OX40 Agonist Potentiates in Vivo Activity. EHA Libr..

[149] FDA Granted Accelerated Approval to Belantamab Mafodotin-Blmf for Multiple Myeloma. https://www.fda.gov/drugs/drug-approvals-and-databases/fda-granted-accelerated-approval-belantamab-mafodotin-blmf-multiple-myeloma.

[150] Avery D.T., Kalled S.L., Ellyard J.I., Ambrose C., Bixler S.A., Thien M., Brink R., Mackay F., Hodgkin P.D., Tangye S.G. (2003). BAFF Selectively Enhances the Survival of Plasmablasts Generated from Human Memory B Cells. J. Clin. Investig..

[151] Chiu A., Xu W., He B., Dillon S.R., Gross J.A., Sievers E., Qiao X., Santini P., Hyjek E., Lee J. (2007). Hodgkin Lymphoma Cells Express TACI and BCMA Receptors and Generate Survival and Proliferation Signals in Response to BAFF and APRIL. Blood.

[152] Novak A.J., Darce J.R., Arendt B.K., Harder B., Henderson K., Kindsvogel W., Gross J.A., Greipp P.R., Jelinek D.F. (2004). Expression of BCMA, TACI, and BAFF-R in Multiple Myeloma: A Mechanism for Growth and Survival. Blood.

[153] Claudio J.O., Masih-Khan E., Tang H., Gonçalves J., Voralia M., Li Z.H., Nadeem V., Cukerman E., Francisco-Pabalan O., Liew C.C. (2002). A Molecular Compendium of Genes Expressed in Multiple Myeloma. Blood.

[154] Galluzzi L., Buqué A., Kepp O., Zitvogel L., Kroemer G. (2017). Immunogenic Cell Death in Cancer and Infectious Disease. Nat. Rev. Immunol..

[155] Haslett P.A., Corral L.G., Albert M., Kaplan G. (1998). Thalidomide Costimulates Primary Human T Lymphocytes, Preferentially Inducing Proliferation, Cytokine Production, and Cytotoxic Responses in the CD8+ Subset. J. Exp. Med..

[156] Davies F.E., Raje N., Hideshima T., Lentzsch S., Young G., Tai Y.-T., Lin B., Podar K., Gupta D., Chauhan D. (2001). Thalidomide and Immunomodulatory Derivatives Augment Natural Killer Cell Cytotoxicity in Multiple Myeloma. Blood.

[157] Schafer P.H., Gandhi A.K., Loveland M.A., Chen R.S., Man H.-W., Schnetkamp P.P.M., Wolbring G., Govinda S., Corral L.G., Payvandi F. (2003). Enhancement of Cytokine Production and AP-1 Transcriptional Activity in T Cells by Thalidomide-Related Immunomodulatory Drugs. J. Pharmacol. Exp. Ther..

[158] Hayashi T., Hideshima T., Akiyama M., Podar K., Yasui H., Raje N., Kumar S., Chauhan D., Treon S.P., Richardson P. (2005). Molecular Mechanisms Whereby Immunomodulatory Drugs Activate Natural Killer Cells: Clinical Application. Br. J. Haematol..

[159] Franssen L.E., Nijhof I.S., Bjorklund C.C., Chiu H., Doorn R., van Velzen J., Emmelot M., van Kessel B., Levin M.-D., Bos G.M.J. (2018). Lenalidomide Combined with Low-Dose Cyclophosphamide and Prednisone Modulates Ikaros and Aiolos in Lymphocytes, Resulting in Immunostimulatory Effects in Lenalidomide-Refractory Multiple Myeloma Patients. Oncotarget.

[160] Hsu A.K., Quach H., Tai T., Prince H.M., Harrison S.J., Trapani J.A., Smyth M.J., Neeson P., Ritchie D.S. (2011). The Immunostimulatory Effect of Lenalidomide on NK-Cell Function Is Profoundly Inhibited by Concurrent Dexamethasone Therapy. Blood.

[161] Fionda C., Abruzzese M.P., Zingoni A., Cecere F., Vulpis E., Peruzzi G., Soriani A., Molfetta R., Paolini R., Ricciardi M.R. (2015). The IMiDs Targets IKZF-1/3 and IRF4 as Novel Negative Regulators of NK Cell-Activating Ligands Expression in Multiple Myeloma. Oncotarget.

[162] Fujiwara Y., Sun Y., Torphy R.J., He J., Yanaga K., Edil B.H., Schulick R.D., Zhu Y. (2018). Pomalidomide Inhibits PD-L1 Induction to Promote Antitumor Immunity. Cancer Res..

[163] Iwasa M., Harada T., Oda A., Bat-Erdene A., Teramachi J., Tenshin H., Ashtar M., Oura M., Sogabe K., Udaka K. (2019). PD-L1 Upregulation in Myeloma Cells by Panobinostat in Combination with Interferon-γ. Oncotarget.

[164] Henry J.Y., Labarthe M.-C., Meyer B., Dasgupta P., Dalgleish A.G., Galustian C. (2013). Enhanced Cross-Priming of Naive CD8 ^+^ T Cells by Dendritic Cells Treated by the IMiDs ^®^ Immunomodulatory Compounds Lenalidomide and Pomalidomide. Immunology.

[165] Galustian C., Meyer B., Labarthe M.-C., Dredge K., Klaschka D., Henry J., Todryk S., Chen R., Muller G., Stirling D. (2009). The Anti-Cancer Agents Lenalidomide and Pomalidomide Inhibit the Proliferation and Function of T Regulatory Cells. Cancer Immunol. Immunother..

[166] Scott G.B., Carter C., Parrish C., Wood P.M., Cook G. (2015). Downregulation of Myeloma-Induced ICOS-L and Regulatory T Cell Generation by Lenalidomide and Dexamethasone Therapy. Cell Immunol..

[167] Hideshima T., Ogiya D., Liu J., Harada T., Kurata K., Bae J., Massefski W., Anderson K.C. (2021). Immunomodulatory Drugs Activate NK Cells via Both Zap-70 and Cereblon-Dependent Pathways. Leukemia.

[168] Fedele P.L., Willis S.N., Liao Y., Low M.S., Rautela J., Segal D.H., Gong J.-N., Huntington N.D., Shi W., Huang D.C.S. (2018). IMiDs Prime Myeloma Cells for Daratumumab-Mediated Cytotoxicity through Loss of Ikaros and Aiolos. Blood.

[169] Chang C.-L., Hsu Y.-T., Wu C.-C., Yang Y.-C., Wang C., Wu T.-C., Hung C.-F. (2012). Immune Mechanism of the Antitumor Effects Generated by Bortezomib. J. Immunol..

[170] Spisek R., Charalambous A., Mazumder A., Vesole D.H., Jagannath S., Dhodapkar M.V. (2007). Bortezomib Enhances Dendritic Cell (DC)–Mediated Induction of Immunity to Human Myeloma via Exposure of Cell Surface Heat Shock Protein 90 on Dying Tumor Cells: Therapeutic Implications. Blood.

[171] Jarauta V., Jaime P., Gonzalo O., de Miguel D., Ramírez-Labrada A., Martínez-Lostao L., Anel A., Pardo J., Marzo I., Naval J. (2016). Inhibition of Autophagy with Chloroquine Potentiates Carfilzomib-Induced Apoptosis in Myeloma Cells in Vitro and in Vivo. Cancer Lett..

[172] Moreno-Bost A., Szmania S., Stone K., Garg T., Hoerring A., Szymonifka J., Shaughnessy J., Barlogie B., Grant Prentice H., van Rhee F. (2011). Epigenetic Modulation of MAGE-A3 Antigen Expression in Multiple Myeloma Following Treatment with the Demethylation Agent 5-Azacitidine and the Histone Deacetlyase Inhibitor MGCD0103. Cytotherapy.

[173] Wu X., Tao Y., Hou J., Meng X., Shi J. (2012). Valproic Acid Upregulates NKG2D Ligand Expression through an ERK-Dependent Mechanism and Potentially Enhances NK Cell-Mediated Lysis of Myeloma. Neoplasia.

[174] Nwangwu C.A., Weiher H., Schmidt-Wolf I.G.H. (2017). Increase of CIK Cell Efficacy by Upregulating Cell Surface MICA and Inhibition of NKG2D Ligand Shedding in Multiple Myeloma: INCREASE OF CIK CELL EFFICACY AGAINST MM. Hematol. Oncol..

[175] García-Guerrero E., Gogishvili T., Danhof S., Schreder M., Pallaud C., Pérez-Simón J.A., Einsele H., Hudecek M. (2017). Panobinostat Induces CD38 Upregulation and Augments the Antimyeloma Efficacy of Daratumumab. Blood.

[176] Song W., Tai Y.-T., Tian Z., Hideshima T., Chauhan D., Nanjappa P., Exley M.A., Anderson K.C., Munshi N.C. (2011). HDAC Inhibition by LBH589 Affects the Phenotype and Function of Human Myeloid Dendritic Cells. Leukemia.

[177] Ray A., Das D.S., Song Y., Hideshima T., Tai Y.-T., Chauhan D., Anderson K.C. (2018). Combination of a Novel HDAC6 Inhibitor ACY-241 and Anti-PD-L1 Antibody Enhances Anti-Tumor Immunity and Cytotoxicity in Multiple Myeloma. Leukemia.

[178] Bae J., Hideshima T., Tai Y.-T., Song Y., Richardson P., Raje N., Munshi N.C., Anderson K.C. (2018). Histone Deacetylase (HDAC) Inhibitor ACY241 Enhances Anti-Tumor Activities of Antigen-Specific Central Memory Cytotoxic T Lymphocytes against Multiple Myeloma and Solid Tumors. Leukemia.

[179] García-Guerrero E., Götz R., Doose S., Sauer M., Rodríguez-Gil A., Nerreter T., Kortüm K.M., Pérez-Simón J.A., Einsele H., Hudecek M. (2020). Upregulation of CD38 Expression on Multiple Myeloma Cells by Novel HDAC6 Inhibitors Is a Class Effect and Augments the Efficacy of Daratumumab. Leukemia.

[180] Bat-Erdene A., Nakamura S., Oda A., Iwasa M., Teramachi J., Ashtar M., Harada T., Miki H., Tenshin H., Hiasa M. (2019). Class 1 HDAC and HDAC 6 Inhibition Inversely Regulates CD 38 Induction in Myeloma Cells via Interferon-α and ATRA. Br. J. Haematol..

[181] Schiavoni G., Sistigu A., Valentini M., Mattei F., Sestili P., Spadaro F., Sanchez M., Lorenzi S., D’Urso M.T., Belardelli F. (2011). Cyclophosphamide Synergizes with Type I Interferons through Systemic Dendritic Cell Reactivation and Induction of Immunogenic Tumor Apoptosis. Cancer Res..

[182] Leong W.I., Ames R.Y., Haverkamp J.M., Torres L., Kline J., Bans A., Rocha L., Gallotta M., Guiducci C., Coffman R.L. (2019). Low-Dose Metronomic Cyclophosphamide Complements the Actions of an Intratumoral C-Class CpG TLR9 Agonist to Potentiate Innate Immunity and Drive Potent T Cell-Mediated Anti-Tumor Responses. Oncotarget.

[183] Buccione C., Fragale A., Polverino F., Ziccheddu G., Aricò E., Belardelli F., Proietti E., Battistini A., Moschella F. (2018). Role of Interferon Regulatory Factor 1 in Governing T Reg Depletion, T H1 Polarization, Inflammasome Activation and Antitumor Efficacy of Cyclophosphamide. Int. J. Cancer.

[184] Rigalou A., Ryan A., Natoni A., Chiu C., Sasser K., O’Dwyer M.E. (2016). Potentiation of Anti-Myeloma Activity of Daratumumab with Combination of Cyclophosphamide, Lenalidomide or Bortezomib Via a Tumor Secretory Response That Greatly Augments Macrophage-Induced ADCP. Blood.

[185] Lorvik K.B., Hammarström C., Fauskanger M., Haabeth O.A.W., Zangani M., Haraldsen G., Bogen B., Corthay A. (2016). Adoptive Transfer of Tumor-Specific Th2 Cells Eradicates Tumors by Triggering an *In Situ* Inflammatory Immune Response. Cancer Res..

[186] Bonanno G., Mariotti A., Procoli A., Folgiero V., Natale D., De Rosa L., Majolino I., Novarese L., Rocci A., Gambella M. (2012). Indoleamine 2,3-Dioxygenase 1 (IDO1) Activity Correlates with Immune System Abnormalities in Multiple Myeloma. J. Transl. Med..

[187] An G., Acharya C., Feng X., Wen K., Zhong M., Zhang L., Munshi N.C., Qiu L., Tai Y.-T., Anderson K.C. (2016). Osteoclasts Promote Immune Suppressive Microenvironment in Multiple Myeloma: Therapeutic Implication. Blood.

[188] Yan H., Dong M., Liu X., Shen Q., He D., Huang X., Zhang E., Lin X., Chen Q., Guo X. (2019). Multiple Myeloma Cell-Derived IL-32γ Increases the Immunosuppressive Function of Macrophages by Promoting Indoleamine 2,3-Dioxygenase (IDO) Expression. Cancer Lett..

[189] Ito S. (2020). Proteasome Inhibitors for the Treatment of Multiple Myeloma. Cancers.

[190] McConkey D.J., Zhu K. (2008). Mechanisms of Proteasome Inhibitor Action and Resistance in Cancer. Drug Resist. Updat..

[191] Imai Y., Hirano M., Kobayashi M., Futami M., Tojo A. (2019). HDAC Inhibitors Exert Anti-Myeloma Effects through Multiple Modes of Action. Cancers.

[192] Ocio E.M., Vilanova D., Atadja P., Maiso P., Crusoe E., Fernández-Lázaro D., Garayoa M., San-Segundo L., Hernández-Iglesias T., de Álava E. (2010). In Vitro and in Vivo Rationale for the Triple Combination of Panobinostat (LBH589) and Dexamethasone with Either Bortezomib or Lenalidomide in Multiple Myeloma. Haematologica.

[193] Swan D., Gurney M., Krawczyk J., Ryan A.E., O’Dwyer M. (2020). Beyond DNA Damage: Exploring the Immunomodulatory Effects of Cyclophosphamide in Multiple Myeloma. Hemasphere.

[194] O’Dwyer M., Henderson R., Naicker S.D., Cahill M.R., Murphy P., Mykytiv V., Quinn J., McEllistrim C., Krawczyk J., Walsh J. (2019). CyBorD-DARA Is Potent Initial Induction for MM and Enhances ADCP: Initial Results of the 16-BCNI-001/CTRIAL-IE 16-02 Study. Blood Adv..

[195] Pham T.-N., Liagre B., Girard-Thernier C., Demougeot C. (2018). Research of Novel Anticancer Agents Targeting Arginase Inhibition. Drug Discov. Today.

[196] Ellyard J.I., Quah B.J.C., Simson L., Parish C.R. (2010). Alternatively Activated Macrophage Possess Antitumor Cytotoxicity That Is Induced by IL-4 and Mediated by Arginase-1. J. Immunother..

[197] Pfeifer S., Schreder M., Bolomsky A., Graffi S., Fuchs D., Sahota S.S., Ludwig H., Zojer N. (2012). Induction of Indoleamine-2,3 Dioxygenase in Bone Marrow Stromal Cells Inhibits Myeloma Cell Growth. J. Cancer Res. Clin. Oncol..

